# HIV-1 intron-containing RNA expression induces innate immune activation and T cell dysfunction

**DOI:** 10.1038/s41467-018-05899-7

**Published:** 2018-08-27

**Authors:** Hisashi Akiyama, Caitlin M. Miller, Chelsea R. Ettinger, Anna C. Belkina, Jennifer E. Snyder-Cappione, Suryaram Gummuluru

**Affiliations:** 1 Department of Microbiology, Boston University School of Medicine, Boston, MA 02118 USA; 2 Department of Pathology, Boston University School of Medicine, Boston, MA 02118 USA; 3 Flow Cytometry Core Facility, Boston University School of Medicine, Boston, MA 02118 USA

## Abstract

Low levels of type I interferon (IFN-I) are thought to be a driving force for immune activation and T-cell exhaustion in HIV-1 infected individuals on combination antiretroviral therapy (cART), though the causative mechanisms for persistent IFN-I signaling have remained unclear. Here, we show Rev–CRM1-dependent nuclear export and peripheral membrane association of intron-containing HIV-1 RNA, independent of primary viral sequence or viral protein expression, is subject to sensing and signaling via MAVS, resulting in IFN-I-dependent pro-inflammatory responses in macrophages. Additionally, HIV-1 intron-containing-RNA-induced innate immune activation of macrophages leads to upregulation of inhibitory receptor expression and functional immune exhaustion of co-cultured T cells. Our findings suggest that persistent expression of HIV-1 intron-containing RNA in macrophages contributes to chronic immune activation and T-cell dysfunction and that use of HIV RNA expression inhibitors as adjunct therapy might abrogate aberrant inflammation and restore immune function in HIV-infected individuals on cART.

## Abstract (web summary)

Type I Interferon is thought to be a driving force for immune activation and T cell exhaustion during HIV infection. Here the authors show that intron-containing HIV RNA induces innate immune activation resulting in associated T cell dysfunction.

## Introduction

A hallmark of HIV-1 infection in vivo is systemic chronic immune activation^1^, which has been postulated to lead to HIV-associated non-AIDS complications (HANA)^2^ and dysfunction of T cells^3^. Despite long-term viral suppression by cART and restoration of CD4^+^ T-cell levels, immune activation, and inflammation persist in the majority of treated HIV-infected individuals, and is associated with excess risk of mortality and morbidity. Many factors have been attributed to cause this aberrant immune activation in vivo, such as bacterial endotoxin or co-infections^4^; however, a viral (HIV) etiology for the chronic inflammatory state has remained unclear. Persistent infection of myeloid cells, most likely tissue-resident macrophages, is postulated to contribute to chronic immune activation and HANAs^5–7^, though molecular mechanisms of how HIV-1 replication activates macrophages remain poorly understood.

In this study, we report that expression and Rev–CRM1-dependent nuclear export of intron-containing HIV-1 RNA (icRNA) activates host sensing mechanisms and type I interferon (IFN-I)-dependent pro-inflammatory responses via MAVS in productively infected macrophages. Additionally, the ability of cells to distinguish intron-containing HIV-1 RNA from self mRNA is dependent on the localization of non-self HIV icRNA at peripheral membrane sites. Interestingly, HIV-1 infection-induced activation of macrophages, in turn, leads to upregulation of inhibitory receptor (IR) expression and reduced effector function of co-cultured autologous CD4^+^ and CD8^+^ T cells, and the phenotype is suppressed upon antagonism of IFN-I. These findings suggest that novel therapeutic strategies that suppress viral icRNA expression and IFN-I signaling cascades in tissue macrophages might have immunologic and therapeutic benefit in HIV-1 infected individuals on cART.

## Results

### Late step of HIV replication triggers MDM immune activation

**Fig. 1:**
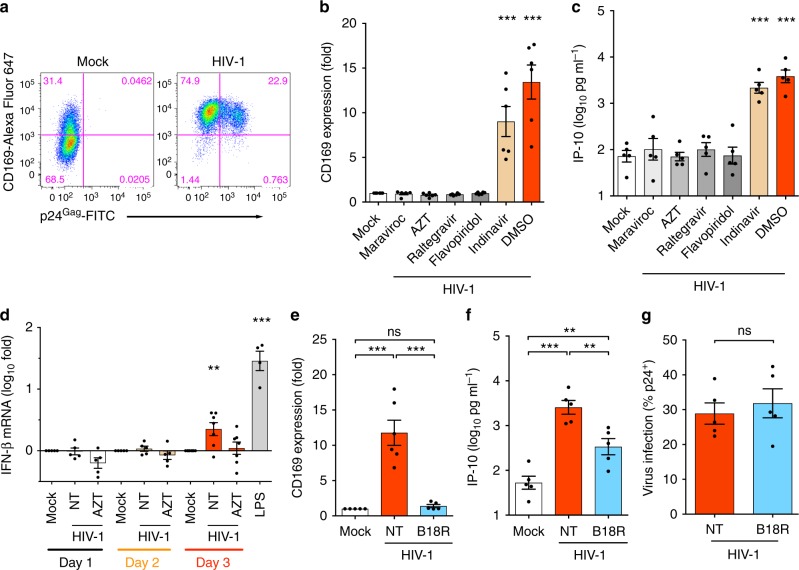
Late step of HIV-1 replication in macrophages triggers immune activation. **a** Flow cytometry profiles (CD169 expression and intracellular p24^Gag^) of MDMs 6 days post infection with replication competent HIV-1 at MOI of 1. **b**, **c** Effects of HIV-1 inhibitors on CD169 expression (**b**) and IP-10 production (**c**) in MDMs. MDMs were treated with drugs prior to infection (maraviroc, AZT or raltegravir) or post infection (flavopiridol and indinavir) with replication competent HIV-1 as described in **a**. **d** Temporal expression of IFN-β mRNA in MDMs. MDMs were infected with a single-round HIV-1 (Lai∆envGFP/G) at MOI of 2 and cells were harvested on day 1, 2, and 3 post infection for mRNA extraction. **e**–**g** Effect of B18R on CD169 expression (**e**), IP-10 production (**f**), and HIV-1 infection (**g**) in MDMs. MDMs were infected with replication competent HIV-1 as in **a** and analyzed 6 days post infection. NT: untreated. The means ± SEM are shown and each symbol represents an independent experiment. Two-tailed *p*-values: one-way ANOVA followed by the Tukey-Kramer post-test (**e**, **f**) or the Dunnett’s post-test (**b**, **c**, and **d**) or paired *t*-test (**g**), *p < 0.05, **p < 0.01, ***p < 0.001, ns: not significant

HIV-1 infection of monocyte-derived macrophages (MDMs) results in induction of a myeloid cell specific ISG, CD169/Siglec1 (Fig. 1a and Supplementary Fig. 1a)^8^ whose expression is dramatically upregulated (fivefold) even upon low levels (<0.3 U ml^–1^) of IFN-α exposure (Supplementary Fig. 1b) in both infected and uninfected bystander MDMs. Interestingly, enhancement of CD169 expression (Fig. 1b and Supplementary Fig. 1c) on MDMs and secretion of pro-inflammatory cytokines, IP-10 (CXCL10) (Fig. 1c), IFN-α2, MCP-1, IL-15, and VEGF (Supplementary Fig. 1d–g) were abrogated upon pre-treatment with inhibitors of HIV-1 fusion (maraviroc), RT (AZT), integration (raltegravir) or p-TEFβ-mediated (i.e., Tat-dependent) transcription (flavopiridol) but not upon treatment with a protease inhibitor (indinavir) (Supplementary Fig. 1h), suggesting that a post-transcriptional step in HIV-1 replication cycle activates MDMs. Furthermore, induction of IFN-β mRNA expression in productively infected MDMs was detected at 3 days post infection (Fig. 1d), which was coincident with the upregulation of CD169 and other ISGs (Supplementary Fig. 1i, j), further supporting the hypothesis that a late event in the virus replication cycle induces IFN-I responses. Moreover, B18R, IFN-I neutralizing reagent, potently inhibited CD169 expression on infected and bystander MDMs (Fig. 1e and Supplementary Fig. 1k) and reduced IP-10 secretion (Fig. 1f), while, co-infection of vesicular stomatitis virus (VSV, whose infection is highly sensitive to IFN-I^9^) was inhibited in HIV-1-infected MDMs (Supplementary Fig. 1l, m), confirming the presence of bioactive IFN-I in the HIV-1-infected MDM culture supernatants. However, the levels of secreted IFN-I were below the detection limit of a conventional bioassay (Supplementary Fig. 1n) and had negligible impact on HIV-1 infection (spread) (Fig. 1g and Supplementary Fig. 1o). Collectively, these results suggest that host sensing of a late step of HIV-1 replication in MDMs induces IFN-I-dependent pro-inflammatory responses.

### HIV structural and accessory proteins do not activate MDM

**Fig. 2:**
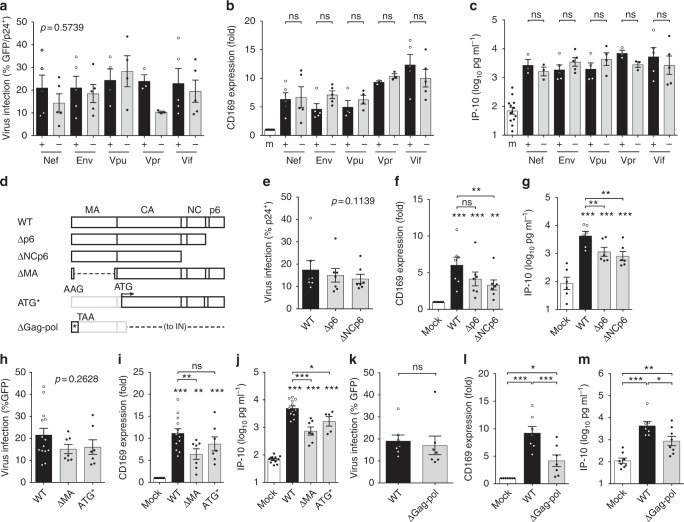
Structural and accessory proteins of HIV-1 do not encode immune activation determinants. **a**–**c** Gag or GFP (viral) expression (**a**), CD169 expression (**b**), and IP-10 production (**c**) in MDMs 6 days post infection with replication competent HIV-1 (Vpu + / −  and Env + ) or VSV-G-pseudotyped single-round viruses (Nef + / - , Env − , Vpr + / − , and Vif + / − ) at MOI of 1. **d** Schematic of HIV-1 Gag mutants. **e**–**m** Gag or GFP (viral) expression (**e**, **h**, **k**), CD169 expression (**f**, **i**, **l**), and IP-10 production (**g**, **j**, **m**) in MDMs 6 days post infection with HIV-1 (MOI of 2). m: mock. The means ± SEM are shown and each symbol represents an independent experiment. Two-tailed *p*-values: one-way ANOVA followed by the Tukey-Kramer post-test (**b**, **c**, **f**, **g**, **i**, **j**, **l** and **m**) and/or the Dunnett’s post-test (**f**, **g**, **i**, and **j**), or paired *t*-test (**k**). *p < 0.05, **p < 0.01, ***p < 0.001, ns: not significant

Although induction of pro-inflammatory responses has been described upon exposure to HIV-1 accessory or structural proteins in diverse cell types^10,11^, MDMs infected with Nef, Env, Vpu, Vpr, or Vif-deficient mutants (Fig. 2a) displayed robust induction of CD169 and IP-10 expression (Fig. 2b, c and Supplementary Fig. 2a). Moreover, MDM infections with Gag p6 (Δp6), NC (ΔNCp6), MA (ΔMA) truncation mutants, or Gag start codon mutant (ATG*) (Fig. 2d, e, h) that results in initiation of Gag translation from an in-frame internal ATG present at the N-terminus of CA (leading to expression of aberrant Gag; Supplementary Fig. 2b) failed to abrogate CD169 or IP-10 induction (Fig. 2f, g, i, j). Additionally, infection of MDMs with HIV-1 budding-deficient mutant (PTAP-)^12^, CA-mutants deficient for Gag–Gag interaction^13^, and cyclophilin A (CyPA)-binding-deficient CA mutant G89V (Supplementary Fig. 2c, f, i) resulted in upregulation of CD169 and IP-10 (Supplementary Fig. 2d, e, g, h, j, k), suggesting that neither virion-release^14^, cytoplasmic accumulation of higher-ordered Gag assembly intermediates nor CyPA-binding to de novo expressed Gag, a target of a cryptic sensor in myeloid dendritic cells^11^, is required for MDM activation. Furthermore, complete abrogation of Gag-pol expression (ΔGag-pol; Fig. 2d and Supplementary Fig. 2b) only modestly attenuated expression of CD169 and IP-10 (Fig. 2l, m) in infected MDMs (Fig. 2k), suggesting that structural and accessory proteins of HIV-1 do not encode immune-activation determinants.

### CRM1-dependent HIV icRNA export is sensed in myeloid cells

**Fig. 3:**
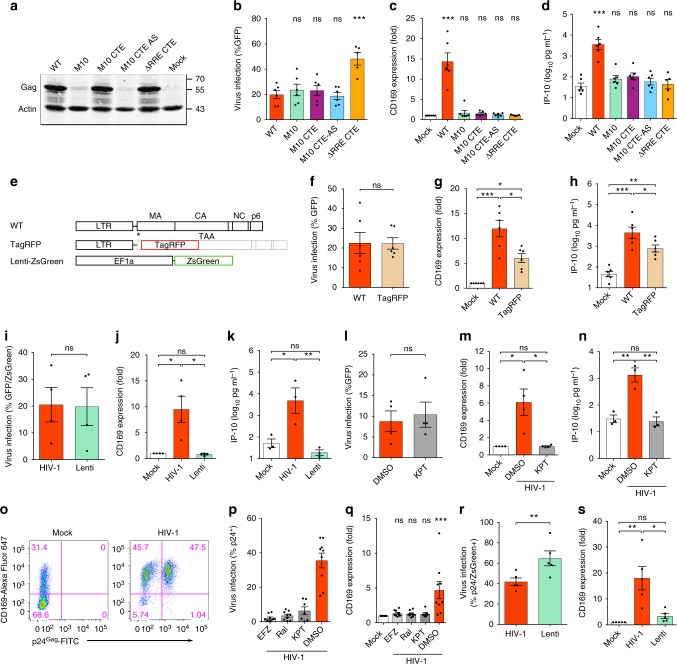
Rev–CRM1-dependent HIV-1 icRNA export is required for sensing. **a**–**d** Effects of Rev/RRE mutations on Gag expression (**a**), GFP (multiply-spliced viral RNA) expression (**b**), CD169 expression (**c**), and IP-10 production (**d**) in MDMs 6 days post infection with HIV-1 (MOI of 2). **e**–**n** Schematic of TagRFP (Rev-dependent) expressing HIV-1 and ZsGreen (Rev-independent) expressing lentivector (**e**), GFP (viral) expression (**f**, **i**), CD169 expression (**g**, **j**), and IP-10 production (**h**, **k**) in MDMs 6 days post infection. **l**–**n** Effects of CRM1 inhibitor (KPT-330) on GFP (viral) expression (**l**), CD169 expression (**m**) and IP-10 production (**n**) in HIV-1 infected MDMs (MOI of 2). **o** Flow cytometry profiles (CD169 expression and intracellular p24^Gag^) of MDDCs infected with replication competent HIV-1 (MOI of 3), 3 days post infection. **p**, **q** Effects of HIV-1 inhibitors and CRM1 inhibitor on intracellular p24^Gag^ expression (viral infection) (**p**) and CD169 expression (**q**) in MDDCs. MDDCs were treated with drugs prior to infection (EFZ: efavirenz or Ral: raltegravir) or post infection (KPT-330) with replication competent HIV-1. **r**, **s** MDDCs were infected with replication competent HIV-1 (MOI of 0.1) or a lentivector expressing ZsGreen (Rev-independent, 100 ng p24^Gag^) in the presence of SIVmac Vpx and extent of infection (intracellular p24^Gag^ or ZsGreen) (**r**) and CD169 expression (**s**) were analyzed on day 3 post infection. The means ± SEM are shown and each symbol represents an independent experiment. Two-tailed *p*-values: one-way ANOVA followed by the Tukey-Kramer post-test (**g**, **h**, **j**, **k**, **m**, **n**, and **s**) or the Dunnett’s post-test (**b**, **c**, **d**, and **q**), or paired *t*-test (**f**, **i**, **l**, and **r**). *p < 0.05, **p < 0.01, ***p < 0.001, ns: not significant

To identify the post-transcriptional viral product that induces pro-inflammatory responses in MDM, we next assessed the effect of viral RNA on MDM activation. There are three classes of viral mRNAs (multiply-spliced, singly-spliced, and unspliced) transcribed from the HIV-1 LTR^15^. Nuclear export of HIV-1 icRNA relies on Rev–RRE binding and a cellular mRNA transporter CRM1^16,17^. Mutations that disrupt Rev binding to CRM1 (M10 mutant; Supplementary Fig. 3a) or delete RRE (∆RRE; Supplementary Fig. 3a), abrogate icRNA nuclear export and thus Gag expression, but not export and translation of multiply-spliced viral RNAs (i.e., GFP in place of *nef*; Fig. 3a, b and Supplementary Fig. 3b)^16,17^. Interestingly, infection of MDMs with the M10 mutant failed to upregulate CD169 and IP-10 expression (Fig. 3c, d). Insertion of the constitutive transporting element (CTE) from Mason-Pfizer monkey virus (MPMV)^18^ within the *pol* open reading frame (orf) of the M10 mutant (M10^-^CTE) or the ∆RRE mutant (∆RRE-CTE) in the sense orientation, but not in the anti-sense orientation (M10-CTE-AS), rescued nuclear export of HIV-1 icRNA and Gag expression (Fig. 3a and Supplementary Fig. 3a, b). However, the rescue of HIV-1 icRNA nuclear export by CTE-dependent pathway failed to induce CD169 or IP-10 expression (Fig. 3c, d). Moreover, while Rev-dependent expression of a non-viral protein (TagRFP) from the intron-containing viral RNA (Fig. 3e, f) induced CD169 and IP-10 expression (Fig. 3g, h and Supplementary Fig. 3c, d), Rev-independent expression of a non-viral protein (ZsGreen) (Fig. 3e, i) from an intron-less mRNA from a lentivector failed to induce CD169 and IP-10 expression (Fig. 3j, k and Supplementary Fig. 3e). A potent CRM1 inhibitor KPT-330 (Selinexor), which prevented icRNA-encoded Gag expression but not multiply-spliced-RNA-encoded GFP (in place of *nef*) expression (Fig. 3l and Supplementary Fig. 3f), potently inhibited upregulation of CD169 (Fig. 3m and Supplementary Fig. 3g) and IP-10 (Fig. 3n), further supporting the importance of Rev–CRM1 pathway in MDM activation.

Myeloid dendritic cells can play an important role in mucosal transmission and systemic dissemination of HIV-1^19,20^. Establishment of productive HIV-1 infection in monocyte-derived dendritic cells (MDDCs) also upregulated CD169 (Fig. 3o), and the activation was suppressed by efavirenz (EFZ, an RT inhibitor), raltegravir (Ral) and KPT-330 (Fig. 3p, q and Supplementary Fig. 3h). However, CD169 expression was not induced in MDDCs by lentivector-driven expression of ZsGreen even at high level of infection (Fig. 3r, s and Supplementary Fig. 3i), phenocopying our findings in MDMs. Collectively, these findings suggest that engagement of divergent RNA nuclear export pathways such as those mediated by Rev–RRE and MPMV CTE might lead to altered exposure of viral icRNA to host cytoplasmic sensing machinery, and that host sensing of viral icRNA specifically exported via Rev–CRM1-dependent pathway is necessary for inducing pro-inflammatory responses in myeloid cells such as MDMs and MDDCs.

### Membrane targeting of HIV icRNA is the trigger for sensing

**Fig. 4:**
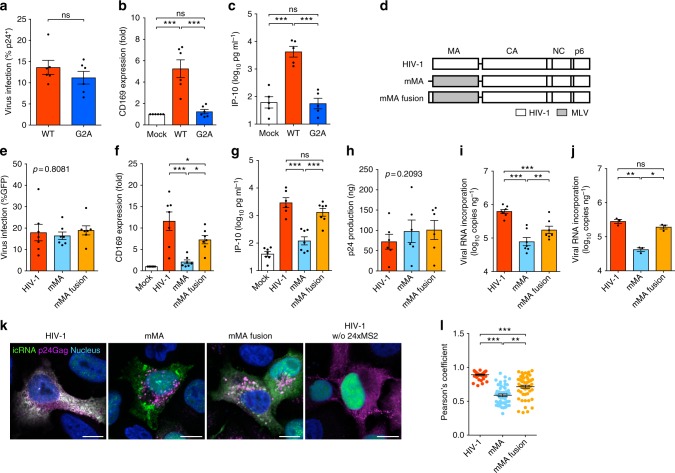
Membrane targeting of viral icRNA is required for MDM activation. **a**–**c** Gag (viral) expression (**a**) CD169 expression (**b**) and IP-10 production (**c**) in wild type (WT) or Gag-G2A-infected (MOI of 2) MDMs on day 6 post infection. **d**–**h** Schematic of MLV/HIV-1 chimeras (**d**) and infection (**e**), CD169 expression (**f**), IP-10 production (**g**), and virion production (**h**) in MDMs. MDMs were infected with HIV-1 mutants at MOI of 1 and examined on day 6 post infection. **i**, **j** Viral RNA incorporation in MDM-derived (**i**) and HeLa-derived (**j**) virions. Virus-containing culture supernatants were harvested on day 6 post infection and virion-associated genomic viral RNA per ng p24^Gag^ was quantified. **k**, **l** Co-localization of p24^Gag^ and intron-containing viral RNA (**k**) and its quantification (**l**) in HeLa. Scale bar: 10 µm. The means ± SEM are shown and each symbol represents an independent experiment. Two-tailed *p*-values: one-way ANOVA followed by the Tukey-Kramer post-test (**b**, **c**, **f**, **g**, **i**, and **j**), a Kruskal–Wallis test followed by the Dunn’s post-test (**l**), or paired *t*-test (**a**). *p < 0.05, **p < 0.01, ***p < 0.001, ns: not significant

HIV-1 icRNA exported via Rev–RRE or CTE-dependent pathway results in distinct cytoplasmic distribution^21^. We, therefore, tested the hypothesis that cytoplasmic localization of HIV-1 icRNA-associated ribonucleoprotein complexes (RNPs) specific to the Rev–RRE pathway dictates induction of pro-inflammatory signaling cascades. Since a subset of cytoplasmic HIV-1 icRNAs are trafficked to membrane-associated virus assembly sites for association with Gag and packaging into new virions^13,15^, we infected MDMs with a series of Gag mutants to potentially modulate localization of HIV-1 icRNA-associated RNPs (Fig. 4a and Supplementary Fig. 4a). Interestingly, infection of MDMs with myristoylation-deficient MA mutant (G2A) that prevents stable membrane association of HIV-1 Gag^13^ attenuated viral icRNA-induced MDM activation (Fig. 4b, c), while none of the other well-characterized MA membrane-targeting domain mutants or those that potentially alter Gag assembly structure or icRNA–Gag association prevented MDM activation (Supplementary Fig. 4b, c). Membrane-targeting function is well conserved among all retroviral MA proteins, though site of virus assembly varies among retroviruses^15^. To putatively alter membrane association sites of HIV-1 icRNA, we introduced diverse lentiviral and retroviral MA sequences in place of HIV-1 MA, including MLV-MA (Fig. 4d). The MLV/HIV chimeric constructs contained the first 42 nucleotides of the HIV-1 *gag* orf followed by the start codon for MLV-MA in order to facilitate optimal incorporation of chimeric viral genome into HIV-1 particles^22^. This additional HIV-1 sequence either contained a point mutation to disrupt the translational initiation codon for HIV-1 Gag sequence such that the chimeric MLV/HIV-1 Gag polyprotein contains no HIV-1 MA residues (mMA, Supplementary Fig. 4d) or retained the intact ATG that leads to translation of a chimeric fusion protein from the intact HIV-1 Gag start codon (mMA-fusion contains the first 14 amino-acid residues of HIV-1 MA, Supplementary Fig. 4d)^22^. Transduction of MDMs with the chimeric MLV/HIV MA viruses revealed that establishment of productive infection with mMA chimeric virus (Fig. 4e) did not induce CD169 or IP-10 production (Fig. 4f, g). Furthermore, complete replacement of the HIV-1 MA with MA and p12 of MLV (Supplementary Fig. 4e), such that the chimeric Gag polyprotein contains no HIV-1 MA sequences^23^, attenuated CD169 and IP-10 expression in productively infected MDMs (Supplementary Fig. 4f–h). In contrast, transduction of MDM with mMA-fusion chimeric virus induced CD169 and IP-10 expression (Fig. 4f, g). Interestingly, MLV-MA-chimeric HIV was unique in its inability to activate MDMs, since infection of MDMs with lentiviruses encoding chimeric Gag with MA sequences derived from other lentiviruses (HIV-2, SIV_mac_, SIV_agm_, SIV_cpz_, SIV_gor_, FIV or EIAV) or deltaretrovirus (HTLVII) (Supplementary Fig. 4i, l) upregulated CD169 and IP-10 expression (Supplementary Fig. 4j, k, m, n). While Gag (p24^CA^) production in mMA and mMA-fusion virus-transduced MDMs was similar to that observed upon infection with wild type HIV-1 (Fig. 4h), mMA virus particles released from MDMs and HeLa cells contained significantly fewer copies of viral genomic RNA compared to HIV-1 (wild type) or mMA-fusion virus particles (Fig. 4i, j), indicative of differential icRNA–Gag association among the three viruses. To investigate localization of viral icRNA in MLV/HIV chimeric Gag expressing cells, viruses were constructed that contained 24 copies of the RNA MS2-binding stem loops in the *pol* orf and encoded a nuclear localized-GFP-bacterial phage MS2 coat fusion protein (MS2-GFP-NLS) located within the *nef* orf (Supplementary Fig. 4o). Since the MS2 stem loops are located in the *pol* orf, only the viral icRNA could be bound and labeled by MS2-GFP proteins (Supplementary Fig. 4o)^24^. We found that while HIV-1 Gag strongly co-localized with the viral icRNA at Gag assembly sites, mMA icRNA and mMA Gag did not co-localize (Fig. 4k, l). Interestingly, co-localization of viral icRNA and Gag was restored in mMA-fusion-expressing cells, though at membrane sites distinct from those observed with wild type HIV-1 (Fig. 4k, l). These results suggest that stable membrane association of viral icRNA is necessary for host sensing and induction of type I IFN-dependent pro-inflammatory signals in MDMs.

### MDM activation is initiated via a MAVS-dependent pathway

**Fig. 5:**
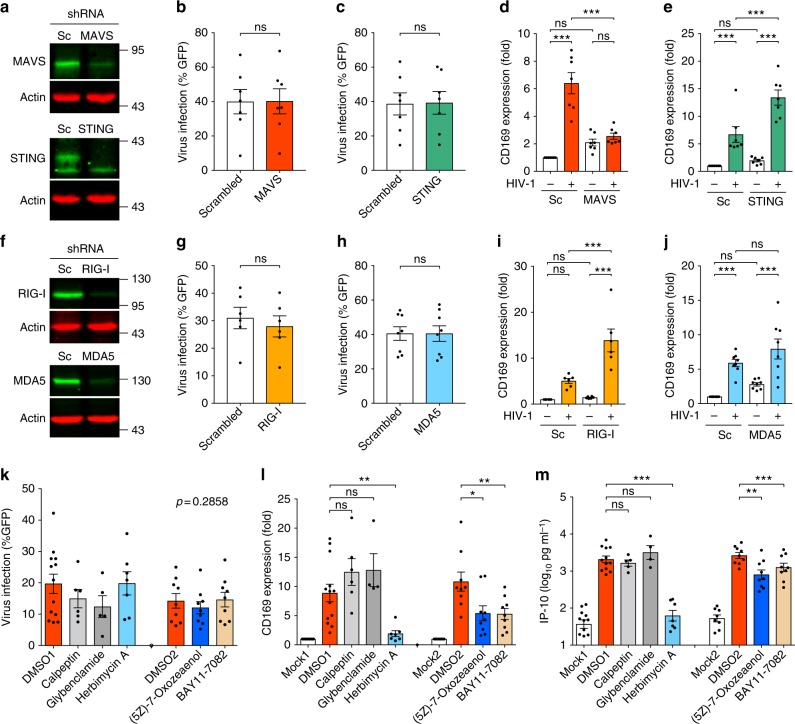
MDM immune activation is initiated via a MAVS-dependent pathway. **a** MDMs were transduced with lentivectors expressing shRNA against scrambled sequence (Sc), MAVS or STING, and the expression of host proteins targeted by shRNAs was analyzed by immune blotting. **b**–**e** shRNA-transduced MDMs were infected with HIV-1 (Lai∆envGFP/G) and viral expression (GFP) (**b**, **c**) and CD169 expression (**d**, **e**) were analyzed 6 days post infection. **f** MDMs were transduced with lentivectors expressing shRNA against scrambled sequence (Sc), RIG-I or MDA5, and the expression host proteins targeted by shRNAs analyzed by immune blotting. **g**–**j** shRNA-transduced MDMs were infected with Lai∆envGFP/G and viral expression (GFP) (**g**, **h**) and CD169 expression (**i**, **j**) were analyzed 6 days post infection. **k**–**m** Effect of inhibitors on HIV-1 infection (**k**), CD169 expression (**l**), and IP-10 production (**m**) in MDMs. MDMs were infected with HIV-1 (MOI of 2) and cultured in the presence of indicated inhibitors for 6 days. mock1/DMSO1: 0.1% DMSO, mock2/DMSO2: 0.01% DMSO. The means ± SEM are shown and each symbol represents an independent experiment. Two-tailed *p*-values: one-way ANOVA followed by the Tukey-Kramer post-test (**d**, **e**, **i**, **j**, **l**, and **m**) or paired *t*-test (**b**, **c**, **g**, and **h**). *p < 0.05, **p < 0.01, ***p < 0.001, ns: not significant

Cytosolic HIV-1-derived nucleic acids can be sensed and trigger induction of innate immune responses via MAVS and/or STING^25^. To identify the signaling pathways induced upon sensing of HIV-1 icRNA, MDMs were transduced with lentivectors expressing shRNA against MAVS or STING (Fig. 5a and Supplementary Fig. 5a). While infection of MDMs containing shRNA against MAVS or STING did not affect subsequent HIV-1 infection (Fig. 5b, c and Supplementary Fig. 5b, c), intriguingly, induction of CD169 expression on MDMs was not observed in the absence of MAVS (Fig. 5d and Supplementary Fig. 5b). In contrast, knockdown of STING expression had a negligible impact on upregulation of CD169 upon HIV-1 infection (Fig. 5e and Supplementary Fig. 5c). Since RIG-I and MDA5 are cell-intrinsic viral nucleic acid sensors that detect cytosolic viral RNAs and trigger MAVS-dependent signaling events^26^, we next investigated whether sensing of HIV-1 icRNA was initiated by RIG-I and MDA5. Knockdown of RIG-I and MDA5 (Fig. 5f and Supplementary Fig. 5a) affected neither susceptibility to HIV-1 infection (Fig. 5g, h and Supplementary Fig. 5d, e) nor enhancement of CD169 expression upon HIV-1 infection in MDMs (Fig. 5i, j and Supplementary Fig. 5d, e), implying there is a yet-to-be identified RNA sensor detecting HIV-1 icRNA.

Since MAVS is thought to provide a mitochondrial membrane-associated scaffold for transducing signaling cascades downstream of numerous pathogen sensing mechanisms^26^, we sought to identify the signaling pathways that activate MAVS and/or are activated via MAVS upon sensing of HIV-1 icRNA. MDMs were infected with single cycle HIV-1 and treated with inhibitors targeting molecules involved in signaling upon pathogen sensing: TAK1 ((5Z)-7-Oxozeaenol)^14^, NF-κB (BAY11-7082)^14^, Ca^2+^-dependent signaling pathway (calpeptin)^27^, NLRP3 inflammasome (glybenclamide)^28^, and herbimycin A, an inhibitor of non-receptor tyrosine kinases including c-Abl, which is involved in MAVS activation^29^. None of the inhibitors were cytotoxic (Supplementary Fig. 5f), and they had negligible effects on HIV-1 infection (Fig. 5k and Supplementary Fig. 5g), Gag expression (Supplementary Fig. 5h), or virus particle release (Supplementary Fig. 5i). We observed that CD169 expression and IP-10 secretion were reduced in infected MDMs upon blocking TAK1 or NF-κB activation and most potently blocked by herbimycin A (Fig. 5l, m and Supplementary Fig. 5g). These results suggest that TAK1, NF-κB, and non-receptor tyrosine kinases are likely involved in MDM activation induced upon host sensing of de novo expressed HIV-1 icRNA.

### HIV-induced MDM activation results in T-cell exhaustion

**Fig. 6:**
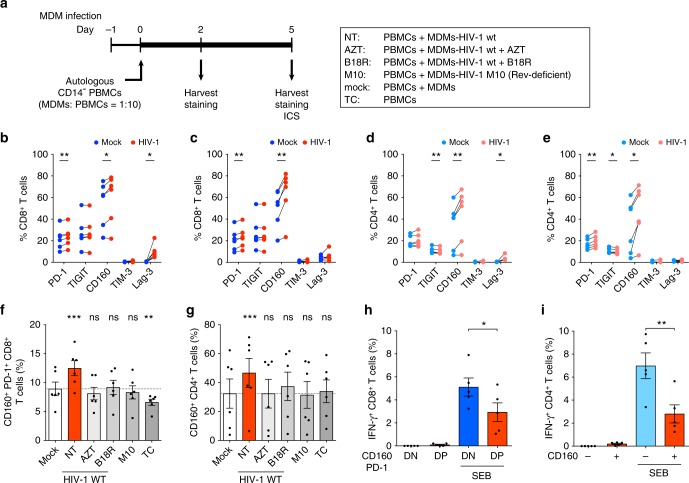
HIV-1-infection-induced MDM activation results in T-cell dysfunction. **a** Schematic of T-cell exhaustion immune phenotyping and intracellular cytokine staining (ICS) assay. MDMs were infected with Lai∆envGFP/G (MOI of 2, wt Rev or M10 Rev) in the presence or absence of AZT or B18R, and autologous CD14^-^ PBMCs were added to MDMs on day 1 post infection (MDM:PBMC ratio = 1:10). **b**–**g** The percentage of IR^+^ CD8^+^ T cells on day 2 (**b**) or day 5 (**c**), or IR^+^ CD4^+^ T cells on day 2 (**d**) or day 5 (**e**) post initiation of co-culture. **f**, **g** The percentage of CD160^+^ PD-1^+^ CD8^+^ T cells (**f**) and CD160^+^ CD4^+^ T cells (**g**) on day 5 post initiation of co-culture. The dotted lines indicate the background levels (mock). **h**, **i** The percentage of IFN-γ producing CD160^+^ PD-1^+^ (DP) or CD160^-^ PD-1^-^ (DN) CD8^+^ T cells (**h**), or CD160^+^ or CD160^-^ CD4^+^ T cells (**i**) in MDM–TC co-culture 6 h post SEB stimulation. The means ± SEM are shown and each symbol represents an independent experiment. Two-tailed *p*-values: one-way ANOVA followed by the Tukey-Kramer post-test (**h**, **i**) or the Dunnett’s post-test (**f**, **g**), or paired *t*-test (**b**–**e**). *p < 0.05, **p < 0.01, ***p < 0.001, ns: not significant

In chronic viral infections such as HIV-1 in humans and lymphocytic choriomeningitis virus (LCMV) in mice, chronic immune activation can result in T-cell exhaustion^3^. Therefore, we sought to determine the effect of MDM immune activation induced by HIV-1 infection on functional capacity of T cells. HIV-1-infected MDMs were co-cultured with autologous CD14-depleted PBMCs, and the expression of IRs, CD160, PD-1, TIM-3, Lag-3 and TIGIT, was measured using flow cytometry (Fig. 6a and Supplementary Fig. 6a). Interestingly, co-culture of HIV-1-infected MDMs and PBMCs upregulated expression of PD-1, CD160, and Lag-3 on CD4^+^ and CD8^+^ T cells (Fig. 6b–e). While Lag-3 expression was transiently enhanced on CD8^+^ T cells (Fig. 6b, c), CD160 expression was upregulated on CD4^+^ and CD8^+^ T cells at both 2 and 5 days post initiation of co-culture (Fig. 6b–e). CD160 expression has been shown to correlate with HIV-1 disease progression and functional impairment of T cells^30,31^. Indeed, we observed increased numbers of CD160^+^ PD-1^+^ CD8^+^ T cells (Fig. 6f) and CD160^+^ CD4^+^ T cells (Fig. 6g) in co-cultures with HIV-infected MDMs, which was not observed when HIV-1 infection in MDMs was inhibited (AZT), soluble IFN-I was neutralized (B18R), or when MDMs were infected with HIV-1/Rev-M10 mutant, which displays a block to HIV-1 icRNA nuclear export (M10). These T-cell exhaustion phenotypes were also seen when co-cultures were initiated with fewer numbers of HIV-infected MDMs (1% GFP^+^ cells at MOI of 0.02) (Supplementary Fig. 6b–d). Finally, we examined functionality of the IR^+^ T cells by performing intracellular cytokine staining for IFN-γ production upon T-cell stimulation with SEB. Intriguingly, we observed a significant decrease in the frequency of IFN-γ producing cells in the CD160^+^ PD-1^+^ double-positive CD8^+^ T-cell population compared to CD160^−^ PD-1^-^ double-negative CD8^+^ T cells (Fig. 6h). Furthermore, a similar decrease in IFN-γ producing cells was observed in CD160^+^ CD4^+^ T cells (Fig. 6i), suggesting that exposure of CD8^+^ and CD4^+^ T cells to HIV-infected MDMs leads to upregulation of IR expression and functional impairment.

## Discussion

Whether HIV-1 infection of MDMs is subject to sensing has remained a matter of controversy. In this study, we show that a late step of HIV-1 replication, specifically, expression and Rev–CRM1-mediated nuclear export of viral icRNA to membrane-associated virus particle assembly sites in MDMs is subject to MAVS-dependent host sensing mechanisms and results in induction of pro-inflammatory responses and innate immune activation. Importantly, Rev–CRM1-dependent nuclear export specifically marks HIV-1 icRNA for detection by cytoplasmic sensing mechanisms, since access to alternative CTE-dependent nuclear export pathway failed to induce pro-inflammatory responses. There seems to be no sequence specificity for the sensing mechanism since inclusion of non-viral sequences (TagRFP) in HIV icRNA failed to abrogate production of pro-inflammatory responses. While the distinct trafficking pattern and composition of viral icRNA-containing RNPs has been hypothesized to confer translational advantage to HIV-1 mRNAs compared to bulk host mRNA^32^, such differences might also subject viral icRNA to detection by host innate immune sensing mechanisms.

It has been shown in neuronal cells that RNA-transporting granules (known as neuronal RNA granules) are trafficked to the vicinity of peripheral membrane sites distant from nucleus, and interestingly many of their components have also been found in HIV-1 RNP trafficking granules^33^. We speculate that HIV-1 icRNA-containing RNPs are by default trafficked to the vicinity of peripheral membrane, and that tethering of HIV-1 icRNA to distinct peripheral membrane sites appeared to be a critical determinant for induction of innate immune sensing. Expression of mutant Gag that alters intrinsic peripheral membrane localization of icRNA (MLV-MA) or by preventing stable membrane association (HIV-1 MA-G2A mutant) abrogated sensing and/or induction of IFN-I responses. Although MAVS requirement for HIV-1-induced MDM activation has been postulated before^34^, viral determinant that was subject to sensing has remained unclear. Here, we identify that Rev–CRM1-dependent icRNA export pathway triggers MAVS-dependent innate immune sensing by a yet-to-be identified sensor that resides at or in the vicinity of HIV-1 Gag assembly site. Further studies are warranted to elucidate HIV-1 icRNA trafficking mechanisms in MDMs, and to identify the cytosolic sensor of HIV-1 icRNA in MDMs.

Expression of several viral proteins, including Gag, Tat and gp120 has been reported to activate myeloid cells including DCs, microglia, and macrophages. For instance, newly synthesized Gag is thought to activate DCs in a cell-intrinsic manner^11^, though results described in this report do not support the existence of a cryptic Gag protein sensor in myeloid cells. Furthermore, previous studies have reported that secreted viral proteins such as gp120 and Tat can cause microglia activation^35^. Differences in experimental system such as target cells and methods to express viral proteins (infections in the presence of SIV_mac_ Vpx to degrade SAMHD1 or transient overexpression) might account for the discrepant findings.

MDM sensing of HIV-1 icRNA expression resulted in low levels of IFN-I production that had negligible effects on virus replication, though the establishment of a IFN-I-dependent chronic inflammatory state might have detrimental consequences to the host. Here, we show that HIV-1-infection-induced MDM activation led to the upregulation of IRs on co-cultured T cells and functional impairment of IR^+^ T cells. Importantly, the observed exhaustion phenotype of T cells was driven by IFN-I since the soluble IFN-I antagonist, B18R, abrogated the upregulation of IR expression on T cells, in agreement with recent in vivo findings on HIV-1^36,37^ and LCMV^38,39^. Tissue-resident macrophages are important cellular targets of HIV and can remain persistently infected with HIV-1 even in individuals on cART^5–7^, and HIV-1 icRNAs have been detected in CD4^+^ T cells and alveolar macrophages from patients on long-term cART^40,41^. A recent study quantified a number of SIV RNA expressing cells in non-human primates on cART and revealed that 98% of those cells were found in gut tissues^42^. While most of the SIV RNA^+^ cells are presumably CD4^+^ T cells, previous estimates suggest myeloid cells including macrophages constitute up to ~35% of total CD45^+^ cells in gut tissues^43^. Although expression of viral RNAs might not lead to functional viral protein production, persistent expression of viral icRNA might trigger chronic innate immune activation in MDMs, that we demonstrate is a potent stimulus of IFN-I-dependent pro-inflammatory responses, contributing to T-cell exhaustion and dysfunction. Although a vast majority of proviruses in HIV^+^ individuals on cART are defective containing large internal deletions and/or APOBEC3G-mediated hypermutations^40,44^, these defective HIV proviruses remain transcriptionally competent encoding non-functional proteins^40^. Future studies will need to investigate whether icRNA expression in myeloid cells is correlated with IR expression in T cells in tissues of HIV/SIV-infected individuals on cART. Unfortunately, current cART regimen does not block virus RNA transcription in previously infected cells. Thus, the development and clinical application of inhibitors that decrease viral RNA expression such as Tat and Rev inhibitors^45,46^, especially in tissue macrophages, could alleviate systemic immune activation in HIV-infected individuals on cART.

## Methods

### Plasmids

HIV-1 replication competent molecular clones, Lai and Lai/YU-2env, single-round reporter constructs, Lai∆env and Lai∆envGFP (GFP in place of the *nef* orf) have been described previously^47,48^. HIV-1 mutants, Lai∆env∆vpr and Lai/YU-2env∆vpu, have been previously reported^47,49^. To create Lai∆envGFP∆vif, a *vif*-containing fragment from Lai∆env-luc∆vif (frame-shift insertions at the *Nde*I site in the *vif* orf)^50^ was replaced into the corresponding region of Lai∆envGFP. Lai∆envGFP∆Gag-pol was created by inserting frame-shift mutations at the *Cla*I site (nt 377) in the Env-expression construct that has a large deletion between N-terminus of CA and IN^47^, and the fragment containing the *env* and *nef* orfs was replaced with the corresponding portion of Lai∆envGFP. The CA G89V R9-GFP mutant and its parental virus R9-GFP were generous gifts from Dr. Christopher R. Aiken (Vanderbilt University School of Medicine). The PTAP- mutant and its parental virus R9 were generously provided by Dr. Wesley I. Sundquist (University of Utah Health). The following Gag mutants were generous gifts from Dr. Jaisri Lingappa (University of Washington): VK181/182AA, K158A, P224A, RS100/102AA, EE75/76AA, ∆p6, ∆NCp6, G2A. mMA12∆envGFP was created by replacing the luciferase gene of MHIV mMA12 (a generous gift from Dr. Masahiro Yamashita, Aaron Diamond AIDS Research Center) with the *gfp* gene. Rev-deficient mutant (M10) was created by PCR-directed mutagenesis using the primer set (Supplementary Table 1). To make RRE-deletion mutant (∆RRE), a *Stu*I-*Ale*I fragment in the *env* orf was deleted from Lai∆envGFP. The CTE was PCR-amplified using the primers (Supplementary Table 1) and pCR4-CTE (a gift from Dr. Mario Chin, Addgene plasmid # 36868) as template and three repeats of CTE were inserted into M10 or ∆RRE between the *Bcl*I (nt 2011) and *Nhe*I (nt 3467) sites in a sense or anti-sense direction. HIV-1 MA-deficient mutants, ∆MA (lacking amino-acid residues 6 to 125) and ATG*, and the following Gag mutants were created by PCR mutagenesis (QuikChangeII, Agilent) or overlapping PCR using the primers listed in Supplementary Table 1: ∆MA, 12LE, 16EK, 21LS, 29/31KE, 30LE, 34VE, 69TR, 85YG, 98EV, 7A2T using Lai∆envGFP as a backbone. To express MA of different viruses, three HIV-1-based vectors were created as follows. Firstly, an *Nco*I site was PCR-generated into at the Gag start codon of Lai∆envGFP (HIV-1-vec, for HIVs, SIVs, and FIV) or at nt 378 (41 nt downstream of Gag starting codon) of Lai∆envGFP (HIV-1-vec-fusion, for mMA-fusion having MLV-HIV fusion MA, see below) or ATG* mutant (HIV-1-vec-plus, for MLV, HTLVII, EIAV, and TagRFP, see below). The latter two vectors have a portion of HIV-1 genome in order to enhance chimeric viral RNA incorporation into virions^22^ with (HIV-vec-fusion) or without (HIV-vec-plus) protein expression from the 41nt HIV-1 sequence. Then, an *Aat*II site was PCR-generated just before the HIV-1 PR cleavage site into all three vectors. *MA* orfs PCR-amplified from HIV-1 Lai, HIV-2 Rod9 (a generous gift from Dr. Michael Emerman, Fred Hutchinson Cancer Research Center), SIVmac239 V1EGFP (a generous gift from Dr. Welkin Johnson, Boston College), SIVagmSab92018ivTF (Drs. Frank Kirchhoff, Clement W. Gnanadurai and Beatrice H. Hahn, NIH AIDS Reagent Program), SIV_CPZ_TAN2.69 (Drs. Jun Takehisa, Matthias H. Kraus, and Beatrice H. Hahn, NIH AIDS Reagent Program), pSIV_gor_CP2139 (Drs. Jun Takehisa, Matthias H. Kraus, and Beatrice H. Hahn, NIH AIDS Reagent Program) and FIV vector (pCPRDEnv, a gift from Dr. Garry Nolan, Addgene plasmid # 1732) were inserted between the above-mentioned *Nco*I and *Aat*II sites of HIV-1-vec. *MA* orfs PCR-amplified from HTLVII plasmid (pH6 B 5.0, Dr. Irvin S.Y. Chen, NIH AIDS Reagent Program), MHIV mMA12, and EIAV vector (pEV53D, a gift from Dr. John Olsen, Addgene plasmid # 44168)^51^ were inserted between the above-mentioned *Nco*I and *Aat*II sites of HIV-1-vec-plus. *MA* orfs PCR-amplified from MHIV mMA12 was also cloned in between the above-mentioned *Nco*I and *Aat*II sites of HIV-1-vec-fusion to create mMA-fusion. TagRFP (Evrogen) was PCR-amplified and inserted into HIV-1-vec-plus. To visualize viral mRNA, the 24xMS2 stem loop cassettes from pCR4-24XMS2SL-stable (a gift from Dr. Robert Singer, Addgene plasmid # 31865)^52^ were inserted between the *Bcl*I (nt 2011) and *Nhe*I (nt 3467) sites in the *pol* orf of HIV-1-vec, mMA (MLA MA in HIV-1-vec-plus, see above) and mMA-fusion. A DNA fragment encoding MS2-GFP-NLS, a fusion protein of the coat protein of bacterial phage MS2 and eGFP-NLS, was PCR-amplified from pMS2-GFP (a gift from Dr. Robert Singer, Addgene plasmid # 27121)^24^ and replaced with the *gfp* gene (in place of *nef*) of HIV-1-vec, mMA, or mMA-fusion with the 24xMS stem loops or Lai∆envGFP having no 24xMS stem loops. All of the mutants were verified by sequencing. A lentivector expressing ZsGreen, pHAGE-ZsGreen, is a generous gift from Dr. Darrell Kotton (Boston University), and HIV-1 packaging plasmid psPAX2, a SIV packaging plasmid containing SIVmac239 Vpx SIV3^+^ and VSV-G expression constructs have been previously described^53^. Lentiviral vectors (pLKO.1) expressing shRNAs were purchased from Sigma or generated by ligating a synthesized double strand DNA into a pLKO.1 cloning vector (provided by Dr. David Root, Addgene plasmid # 10878) digested with *Age*I and *Eco*RI.

### Cells

Human monocyte-derived macrophages (MDMs) and monocyte-derived dendritic cells (MDDCs) were derived from beads-isolated CD14^+^ peripheral blood monocytes^47^ by culturing in RPMI1640 (Invitrogen) containing 10% heat-inactivated human AB serum (Gemini Bio Products, Sigma or Corning) and recombinant human M-CSF (20 ng ml^–1^; Peprotech) or containing 10% heat-inactivated fetal bovine serum supplemented with human GM-CSF (10 ng ml^–1^, Miltenyi) and IL-4 (1000 U  ml^–1^, BD), respectively, for 5–6 days. HEK293T (ATCC), 293 ISRE-luc (Drs. Junzhi Wang and Xuguang Li), HeLa (NIH AIDS Reagent Program) and TZM-bl (NIH AIDS Reagent Program) were maintained in DMEM (Invitrogen) containing 10% heat-inactivated FBS (Invitrogen)^49,53,54^. All cell lines have been tested for mycoplasma contamination and confirmed negative. Cell viability was measured by a conventional MTT assay.

### Viruses

Replication competent viruses were derived from HEK293T cells via calcium phosphate transient transfection^53^. Single-round-replication competent viruses pseudotyped with VSV-G were generated from HEK293T cells via co-transfection of HIV-1∆env constructs and VSV-G expression plasmid. To express HIV-1 mutants which by themselves cannot infect MDMs, a mutant plasmid was co-transfected with the packaging construct (psPAX2) and a VSV-G expression vector into HEK293T^53^. Lentivector expressing ZsGreen or shRNA was also generated by co-transfecting HEK293T cells with psPAX2 and a VSV-G expression vector using calcium phosphate or TransIT-293 (Mirus). Virus-containing cell supernatants were harvested 2 days post-transfection, cleared of cell debris by centrifugation (300 x *g*, 5 min), passed through 0.45 µm filters, and purified and concentrated by ultracentrifugation on a 20% sucrose cushion (24,000 rpm and 4 °C for 2 h with a SW32Ti or SW28 rotor (Beckman Coulter)). The virus pellets were resuspended in PBS, aliquoted, and stored at −80 °C until use. The capsid content of HIV-1 was determined by a p24^gag^ ELISA^53^ and virus titer was measured on TZM-bl^55^. VSV expressing dsRed upon infection was kindly provided by Dr. John Connor (Boston University).

### Infection

MDMs seeded 1 day before infection or MDDCs were spinoculated with viruses (1 h at room temperature (RT) and 2300 rpm) in the presence of polybrene (Milipore) at various multiplicity of infection (MOI, typically 0.5 to 2 for MDMs and 0.1 to 3 for MDDCs), cultured for 2–3 h at 37 °C, washed to remove unbound virus particles, and cultured for 3–6 days. Infection in MDMs or MDDCs was quantified by analyzing intracellular p24^Gag^ or GFP expression by flow cytometry. In some experiments, MDMs or MDDCs were pretreated prior (at least 30 min) to infection with maraviroc (20 nM, NIH AIDS Reagent Program), AZT (5 µM, NIH AIDS Reagent Program), efavirenz (1 µM, NIH AIDS Reagent Program), raltegravir (30 µM, NIH AIDS Reagent Program or Selleckchem), or treated 2–3 h post infection with flavopiridol (100 nM, NIH AIDS Reagent Program), indinavir (2 µM, NIH AIDS Reagent Program), B18R (1 µg ml^–1^, eBioscience), calpeptin (30 µg ml^–1^, Calbiochem), glybenclamide (50 µM, InvivoGen), (5Z)-7-Oxozeaenol (1 µM, Calbiochem), BAY11-7082 (5 µM, InvivoGen), herbimycin A (10 µM, Tocris or Cayman Chemical), and KPT-330 (200 nM or 1 µM, Selinexor, Selleckchem).

### shRNA-mediated knockdown

To knockdown expression of host proteins, MDMs were transduced with lentivectors expressing shRNA (pLKO.1, 400 ng p24^Gag^ content per 1 × 10^6^ cells) in the presence of SIVmac Vpx-containing VLPs pseudotyped with VSV-G (SIV3^+^/G) and polybrene (Millipore) by spinoculation as described above. After culturing for 2 days, MDMs were cultured for another 5–7 days in the presence of puromycin (3 µg ml^–1^, GIBCO or InvivoGen) for selection before being used for infection or other experiments. Knockdown efficiency was quantified by qRT-PCR or western blotting. All the shRNA target sequences are listed in Supplementary Table 2.

### Immunoblot analysis

To assess expression of viral proteins (p24 and p17) or to validate knockdown of host proteins (MAVS, STING, RIG-I, and MDA5), cell lysates containing 20−30 µg total protein were separated by sodium dodecyl sulfate–polyacrylamide gel electrophoresis, transferred to nitrocellulose membranes and the membranes were probed with the following antibodies: a mouse anti-p24 antibody (p24-2, Dr. Michael H. Malim, NIH AIDS Reagent Program, #6457, 1:5000) or a rabbit anti-p24 antibody (ImmunoDX, #1303, 1:1000), a rabbit anti-p17 polyclonal antibody (VU47, Dr. Paul Spearman and Dr. Lingmei Ding, NIH AIDS Reagent Program, #4811, 1:1000), a rabbit anti-MAVS polyclonal antibody (Thermo Fisher, #PA5-17256, 1:1000), a rabbit anti-STING polyclonal antibody (Cell Signaling, #13647, 1:1000), a mouse anti-RIG-I antibody (Enzo Life Sciences, #ALX-804-849-C100, 1:1500) or a rabbit anti-MDA5 antibody (Proteintech, #21775-1-AP, 1:1000). Then, the membranes were stained with secondary antibodies, a goat anti-mouse-IgG-DyLight 680 (Pierce, # 35518, 1:10,000) or a goat anti-rabbit-IgG-DyLight 800 (Pierce, # 35571, 1:10,000). As loading controls, actin was probed using a rabbit anti-actin antibody (SIGMA, # A2066, 1:5000) or a mouse anti-actin antibody (Thermo Fisher, #AM4302, 1:5000). Membranes were scanned with an Odessy scanner (Li-Cor). All uncropped images are shown in Supplementary Fig. 7.

### mRNA and viral RNA Quantification

Total mRNA was isolated from 1 × 10^6^ cells using a kit (RNeasy kit, QIAGEN) and reverse-transcribed using oligo(dT)_20_ primer (Superscript III, Invitrogen). Target mRNA was quantified using Maxima SYBR Green (Thermo Scientific) and normalized to GAPDH mRNA by the 2^-∆∆*C*^_T_ method as described^49,56^. As a positive control for IFN-β mRNA expression, MDMs were treated with 1 µg ml^–1^ LPS (InvivoGen) for 2 h. For virion-associated viral RNA quantification, viral RNA was isolated from 140 µl cell-free culture supernatants using Qiamp Viral RNA mini kit (QIAGEN) and reverse-transcribed using random hexamers (Superscript III, Invitrogen). Viral cDNA was then quantified using Maxima SYBR Green (Thermo Scientific) using pLai∆envGFP as standard. All the primers used for quantitative PCR are listed in the Supplementary Table 3.

### Cytokine quantification

IP-10 in MDM culture supernatants was measured with a BD Human IP-10 ELISA Set (BD). Multiplex cytokine assay using a human cytokine 29-plex kit (Millipore) was performed using culture supernatants and was analyzed with a DropArray (Curiox Biosystems) and the MAGPIX System (Luminex). Bioactive IFN-I was measured by a bioassay using 293 ISRE-luc cell line (a gift from Drs. Junzhi Wang, National Institute for the Control of Pharmaceutical and Biological Products, China and Xuguang Li, University of Ottawa, Canada)^49^.

### Flow cytometry

CD169 expression on MDMs was quantified by staining with Alexa647-conjugated mouse anti-human CD169 antibody (BioLegend, # 346006, 1:50) and analyzed with BD LSRII (BD). Geo mean fluorescence intensity (MFI) was calculated and normalized to that of uninfected (mock) MDMs. In some experiments, intracellular p24 was stained as described^49^ using FITC- or RD1-conjugated mouse anti-p24 monoclonal antibody (KC57, Coulter, # 6604665 or # 6604667, 1:25). For T-cell exhaustion assay, MDM–PBMC co-culture was harvested, stained with Zombie-NIR (BioLegend, #423105, 1:250) and a panel of makers designed for inhibitory receptor expression profiling^57^, and analyzed with FACS ARIA (BD). For intracellular IFN-γ staining, MDM–PBMC co-culture was unstimulated or stimulated with SEB (List Biological Laboratories) for 1 h, followed by culture with brefeldin A (BioLegend) for another 5 h, stained for inhibitory receptors as described above, fixed with FluoroFix Buffer (BioLegend), and stored in a staining buffer (2% NCS in PBS). Fixed cells were permeabilized with intracellular staining permeabilization wash buffer (BioLegend), stained with anti-human IFN-γ PE (BioLegend, # 506506, 1:200), and analyzed with FACS ARIA (BD). Data was analyzed with FlowJo software (FlowJo).

### Imaging

HeLa cells seeded on coverslips were transfected with a plasmid containing 24xMS2 binding loops and MS2-GFP-NLS by lipofectamine 2000 (Invitrogen). Cells were washed and fixed 24 h post-transfection with 4% parafolmaldehyde, permeabilized with 0.1% TritonX100, and stained with mouse anti-p24 antibody (AG3.0, Dr. Jonathan Allan, NIH AIDS Reagent Program, # 4121, 1:250) followed by staining with Alexa594-conjugated anti-mouse-IgG antibody (Invitrogen, # A-11020, 1:200) and DAPI (Sigma). Cells were mounted on a slide glass with Fluoromount-G (Southern Biotech) and imaged with a confocal microscopy (Leica SP5). For co-localization analysis, more than 50 cells were imaged, and correlation between p24 signals and GFP signals in cytosol (DAPI negative) were analyzed with ImageJ (NIH).

### Statistics

All the statistics analysis was performed using GraphPad Prism 5. Two-tailed *p*-values were calculated using one-way ANOVA followed by the Tukey-Kramer post-test (symbols for *p*-values shown with a line) or the Dunnett’s post-test (comparing to control (mock or wild type), symbols for *p*-values shown on each column), a Kruskal–Wallis test followed by the Dunn’s post-test (symbols for *p*-values shown with a line), or a paired *t*-test (comparing two samples, symbols for *p*-values shown with a line). Symbols represent, *p < 0.05, **p < 0.01, ***p < 0.001, ns: not significant (*p* ≥ 0.05).

The numbers of experimental replicates (*n*) and statistical information of each figure are as follows. Figure 1a, repeated > 10 times, Fig. 1b: *n* = 6, Fig. 1c: *n* = 5; Fig. 1d: Day 1 *n* = 5, Day 2 *n* = 5, Day 3 *n* = 7, LPS *n* = 4; Fig. 1e, f, *n* = 5; Fig. 1g, *n* = 5, *t* = 1.815 degrees of freedom (df) = 4. Figure 2a, *n*: + / -  Nef 5, + / -  Env 5, + / -  Vpu 4, + / -  Vpr 3, + / -  Vif 5, *F* = 0.8536; Fig. 2b, *n*: mock 17: + / -  Nef 5, + / -  Env 5, + / -  Vpu 4, + / -  Vpr 3, + / -  Vif 5; Fig. 2c, n: mock 13, + / -  Nef 3, + / -  Env 5, + / -  Vpu 4, + / -  Vpr 3, + / -  Vif 5; Fig. 2e, *n* = 7, *F* = 2.618, df = 20; Fig. 2f, *n* = 7; Fig. 2g, *n* = 6; Fig. 2h, *n* = 5, *F* = 1.411, df = 27, *n*: WT 14, ∆MA 7, ATG* 7; Fig. 2i, *n*: mock 14, WT 13, ∆MA 7, ATG* 7; Fig. 2j, *n*: mock 12, WT 12, ∆MA 7, ATG* 5; Fig. 2k, *n* = 7, *t* = 0.9510, df = 6; Fig. 2l, *n* = 7, Fig. 2m, *n*: mock 8, WT 7, ∆Gag-pol 8. Figure 3a, *n* = 4; Fig. 3b, *n*: WT/M10/M10 CTE/M10 CTE-AS: 6, ΔRRE-CTE: 5; Fig. 3c, d, *n*: mock/WT/M10/M10 CTE/M10 CTE-AS = 6, ΔRRE-CTE = 5; Fig. 3f, *n* = 6, *t* = 0.07817, df = 5; Fig. 3g, h, *n* = 6; Fig. 3i, *n* = 4, *t* = 0.3356, df = 3; Fig. 3j, *n* = 4; Fig. 3k, *n* = 3; Fig. 3l, *n* = 4, *t* = 0.6931, df = 3; Fig. 3m, *n* = 4; Fig. 3n, *n* = 3; Fig. 3o, *n* = 9; Fig. 3p, q, *n*: KPT = 7, mock/EFZ/Ral/DMSO = 9; Fig. 3r, *n* = 5, *t* = 4.933, df = 4; Fig. 3s, *n* = 5. Figure 4a, *n* = 6, *t* = 1.339, df = 5; Fig. 4b, *n* = 6; Fig. 4c, *n* = 5; Fig. 4e, *n* = 7, *F* = 0.2156, df = 20; Fig. 4f, *n* = 7; Fig. 4g, *n*: HIV-1 = 6, mock/mMA/mMA-fusion = 7; Fig. 4h, *n* = 6, *F* = 1.837, df = 17; Fig. 4i, *n* = 7; Fig. 4j, *n* = 3; Fig. 4k–l, *n*: HIV-1/mMA = 50, mMA-fusion = 53. Figure 5a, *n*: MAVS = 4, STING = 3; Fig. 5b, *n* = 7, *t* = 0.1929, df = 6; Fig. 5c, *n* = 7, *t* = 0.6252, df = 6; Fig. 5d, e, *n* = 7; Fig. 5f, *n*: RIG-I = 4, MDA5 = 3; Fig. 5g, *n* = 6, *t* = 2.516, df = 5; Fig. 5h, *n* = 8, *t* = 0.05451, df = 7; Fig. 5i, *n* = 6; Fig. 5j, *n* = 8; Fig. 5k, *n*: DMSO1 = 13, (5Z)-7-Oxozeaenol/BAY11-7082/ DMSO2 = 9, Herbimycin *A* = 7, Calpeptin = 6, Glybenclamide = 5, *F* = 1.274, df = 57; Fig. 5l, *n*: DMSO1 = 13, (5Z)-7-Oxozeaenol/BAY11-7082/ DMSO2 = 9, Herbimycin *A* = 7, Calpeptin = 6, Glybenclamide = 4; Fig. 5l, *n*: DMSO1 = 12, (5Z)-7-Oxozeaenol/BAY11-7082/ DMSO2 = 9, Herbimycin *A* = 7, Calpeptin = 5, Glybenclamide = 4. Figure 6b–g, *n* = 6; Fig. 6h, i, *n* = 5. Supplementary Fig. 1: 1a, *n* = 6; 1b, *n* = 3; 1c, *n* = 6; 1d–g, *n* = 5; 1h, i, *n* = 6; 1j, *n* = 3; 1k, *n* = 6; 1 l, m, *n* = 4; 1n, o, *n* = 6. Supplementary Figure 2: 2a, *n*: mock 17: + / -  Nef 5, + / -  Env 5, + / -  Vpu 4, + / -  Vpr 3, + / -  Vif 5; Fig. 2c, *n*: mock 13; 2b, *n* = 3; 2c, *n* = 5, *t* = 0.3200, df = 4; 2d, *n* = 5; 2e, *n* = 4; 2f, g, *n* = 6; 2 h, *n* = 5; 2i, *n* = 5, *t* = 0.3056, df = 4; 2j, *n* = 5; 2k, *n* = 4. Supplementary Fig. 3: 3b, *n* = 4; 3c, d, *n* = 6; 3e, *n* = 4; 3f, g, *n* = 4; 3 h, *n* = 9; 3i, *n* = 5. Supplementary Fig. 4: 4a, *n*: WT = 7, 29/31KE/85YG/16EK/12LE/30LE/98EV = 5, 7A2T/21LS/34VE/69TR = 4, *F* = 0.5085, df = 50; 4b, *n*: WT = 7, 29/31KE/85YG/16EK/12LE/30LE/98EV = 5, 7A2T/21LS/34VE/69TR = 4, mock = 8; 4c, *n*: WT = 9, 29/31KE/85YG/16EK/12LE/30LE/98EV = 5, 7A2T/21LS/34VE/69TR = 4, mock = 9; 4f, *n* = 6, *t* = 0.3934, df = 5; 4g, h, *n* = 6; 4i, n: HIV-1 = 8, HIV-2 = 7, SIVmac/SIVagm/SIVcpz/SIVGor = 5, *F* = 0.2291, df = 34; 4j, k, *n*: HIV-1/mock = 8, HIV-2 = 7, SIVmac/SIVagm/SIVcpz/SIVGor = 5; 4 l, *n*: HIV-1 = 17, FIV = 6, HTLVII = 8, EIAV = 9; 4 m, *n*: HIV-1 = 15, FIV = 6, HTLVII = 8, EIAV = 9, mock = 20; 4n, *n*: HIV-1 = 9, FIV = 6, HTLVII = 8, EIAV = 9, mock = 13. Supplementary Fig. 5: 5a, *n*: Scrambled = 18, MAVS/STING = 7, RIG-I = 5, MDA5 = 8; 5b, c, *n* = 7; 5d, *n* = 6; 5e, *n* = 8; 5f, *n* = 3; 5g, *n*: mock/DMSO = 13, (5Z)-7-Oxozeaenol/BAY11-7082 = 9, Herbimycin *A* = 7, Calpeptin = 6, Glybenclamide = 4; 5 h, *n* = 5; 5i, *n*: DMSO2/(5Z)-7-Oxozeaenol/BAY11-7082 = 6, Herbimycin A/DMSO1 = 5, *F* = 0.3910, df = 27. Supplementary Fig. 6b–d, *n* = 3.

## Electronic supplementary material

Supplementary Information

## Data Availability

The authors declare that the data that support the findings of this study are available within the paper and from the corresponding authors upon reasonable request.
