# Supplementary material for: HIV-1 intron-containing RNA expression induces innate immune activation and T cell dysfunction
Source: Nat Commun. 2018 Aug 27;9:3450. doi: 10.1038/s41467-018-05899-7 (PMC6110775; doi:10.1038/s41467-018-05899-7)
Supplement: Supplementary file 1 — Supplementary Information [file 41467_2018_5899_MOESM1_ESM.pdf]

Supplementary Information

HIV-1 Intron-containing RNA Expression Induces Innate Immune Activation and T Cell Dysfunction

Akiyama et al.

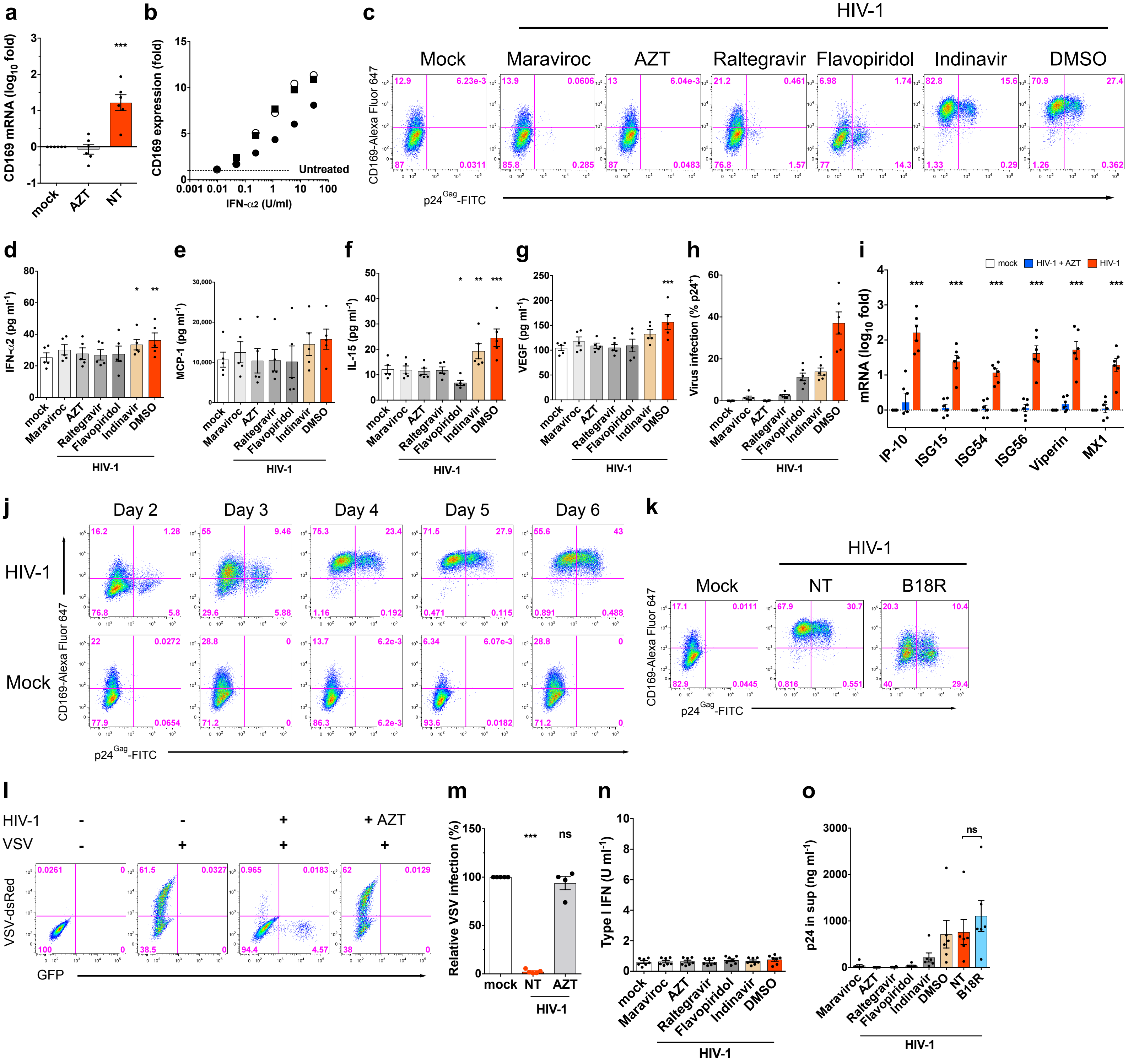

Supplementary Figure 1. Late step of HIV-1 replication in MDMs triggers immune activation.
a, MDMs were infected with LaiΔenvGFP/G (MOI 2) and harvested for mRNA isolation 3 days post infection (dpi). CD169 mRNA was quantified by qRT-PCR and normalized to that of mock. **b**, MDMs were incubated with the indicated amounts of IFN-α_2 for 2 days, and CD169 expression was measured by flow cytometry. **c**, Representative flow cytometry profiles of MDMs infected with Lai/YU-2env (MOI 1) and analyzed for viral infection (intracellular $p24^{Gag}$) and CD169 expression 6 dpi. MDMs were untreated (DMSO) or treated with drugs prior to infection (maraviroc, AZT or raltegravir), or 2-3 hours post infection (flavopiridol or indinavir). **d-g**, Cytokine production in HIV-1-infected MDMs was measured by Luminex (6 dpi). **h**, HIV-1 infection in MDMs was measured by intracellular $p24^{Gag}$ staining. **i**, ISG expression in MDMs infected with LaiΔenvGFP/G (MOI 2) was quantified by qRT-PCR and normalized to that of mock (3 dpi). **j**, Kinetics of CD169 expression. Uninfected MDMs and MDMs infected with Lai/YU-2env (MOI 1) were analyzed 2 to 6 dpi for CD169 and HIV-1 infection (intracellular $p24^{Gag}$). Representative flow cytometry profiles are shown. **k**, Representative flow cytometry profiles of MDMs infected with Lai/YU-2env (MOI 1) in the presence of B18R (6 dpi). **l**, VSV infection in HIV-1-infected MDMs. MDMs were uninfected (mock) or infected with HIV-1 (GFP in place of *nef*) in the absence (NT) or presence of AZT for 6 days, and further infected with VSV-dsRed for 16 hours. **m**, The percentage of VSV infection was normalized to that of mock. **n**, IFN-I secretion from HIV-1-infected MDMs (6 dpi) was measured by a bioassay. **o**, Virus production ($p24^{Gag}$) in HIV-1-infected MDMs culture supernatants was measured by ELISA (6 dpi). The data shown are the means ± SEM and each symbol represents data obtained from cells derived from an independent donor. Two-tailed p values were calculated using one-way ANOVA followed by the Dunnett's post-test (**a, d-h and m**) or the Tukey-Kramer post-test (**o**). *: $p < 0.05$, **: $p < 0.01$, ***: $p < 0.001$, ns: not significant.

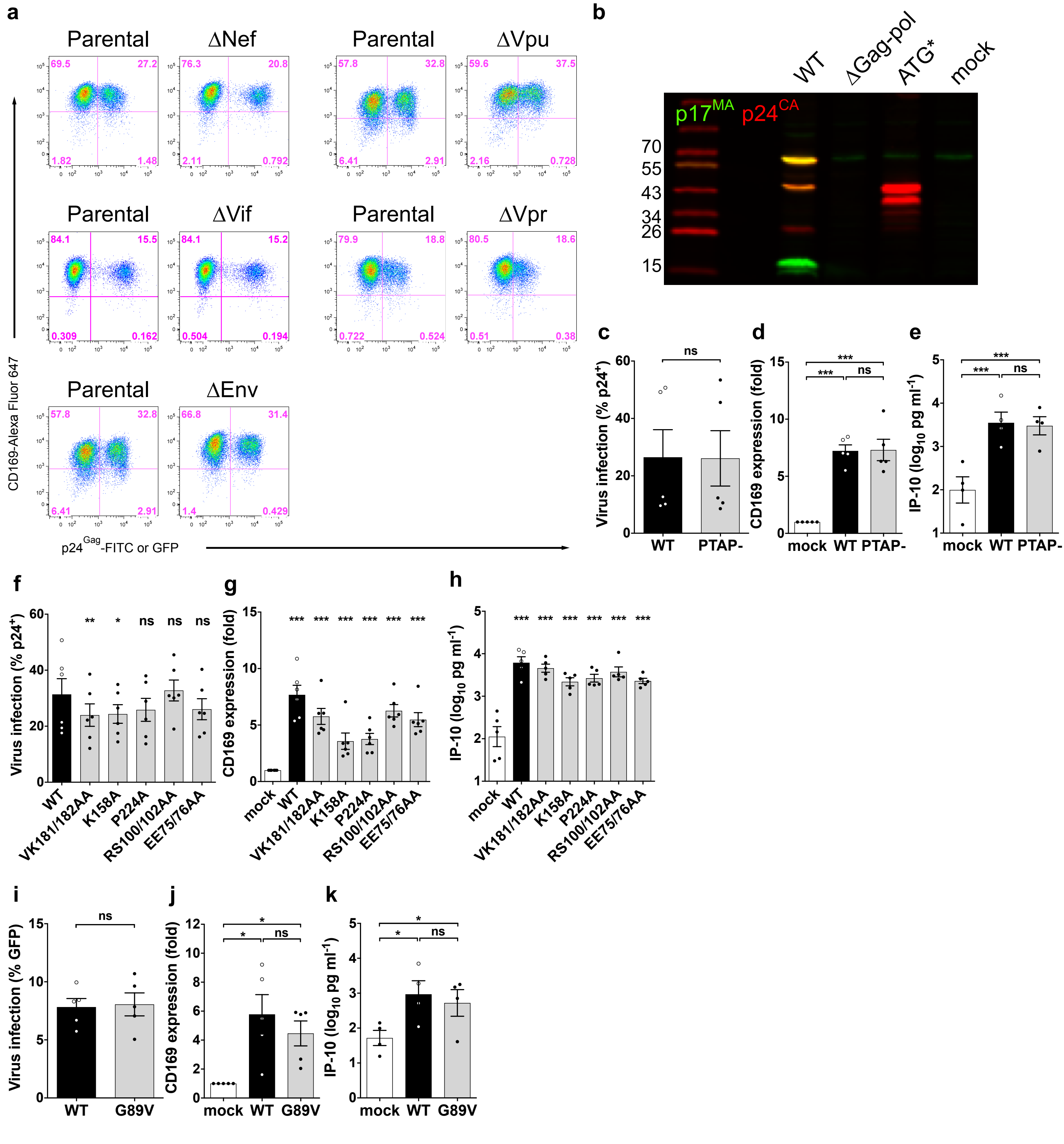

Supplementary Figure 2. Structural and accessory proteins of HIV-1 do not encode immune-activation determinants.
a, Representative flow cytometry profiles of MDMs infected with HIV-1 mutants and their parental clones at MOI of 1 and analyzed on 6 days post infection for CD169 and viral infection (intracellular $p24^{Gag}$ staining or GFP expression) (6 dpi). **b**, Western blot analysis for MA (p17) and CA (p24) expression in HEK293T cells transfected with LaiΔenvGFP (WT), ΔGag-pol mutant, or ATG* mutant. **c**-**e**, HIV-1 expression (intracellular $p24^{Gag}$) (**c**), CD169 expression (**d**), and IP-10 production (**e**) in MDMs transduced with the wild type (WT) or PTAP- mutant (6 dpi). **f**-**h**, HIV-1 expression (intracellular $p24^{Gag}$) (**f**), CD169 expression (**g**), and IP-10 production (**h**) in MDMs transduced with the wild type (WT) or indicated CA mutants deficient for intra- or inter-hexamer formation (6 dpi). **i**-**k**, HIV-1 expression (GFP) (**i**), CD169 expression (**j**), and IP-10 production (**k**) in MDMs transduced with the wild type (WT) or CyPA-binding-deficient mutant (G89V) (6 dpi). The data shown are the means ± SEM and each symbol represents data obtained from cells derived from an independent donor. Two-tailed p values were calculated using one-way ANOVA followed by the Tukey-Kramer post-test (**d**, **e**, **j**, **k**) or the Dunnett's post-test (**f**, **g**, **h**), or a paired t-test (**c**, **i**). *: $p < 0.05$, **: $p < 0.01$, ***: $p < 0.001$, ns: not significant.

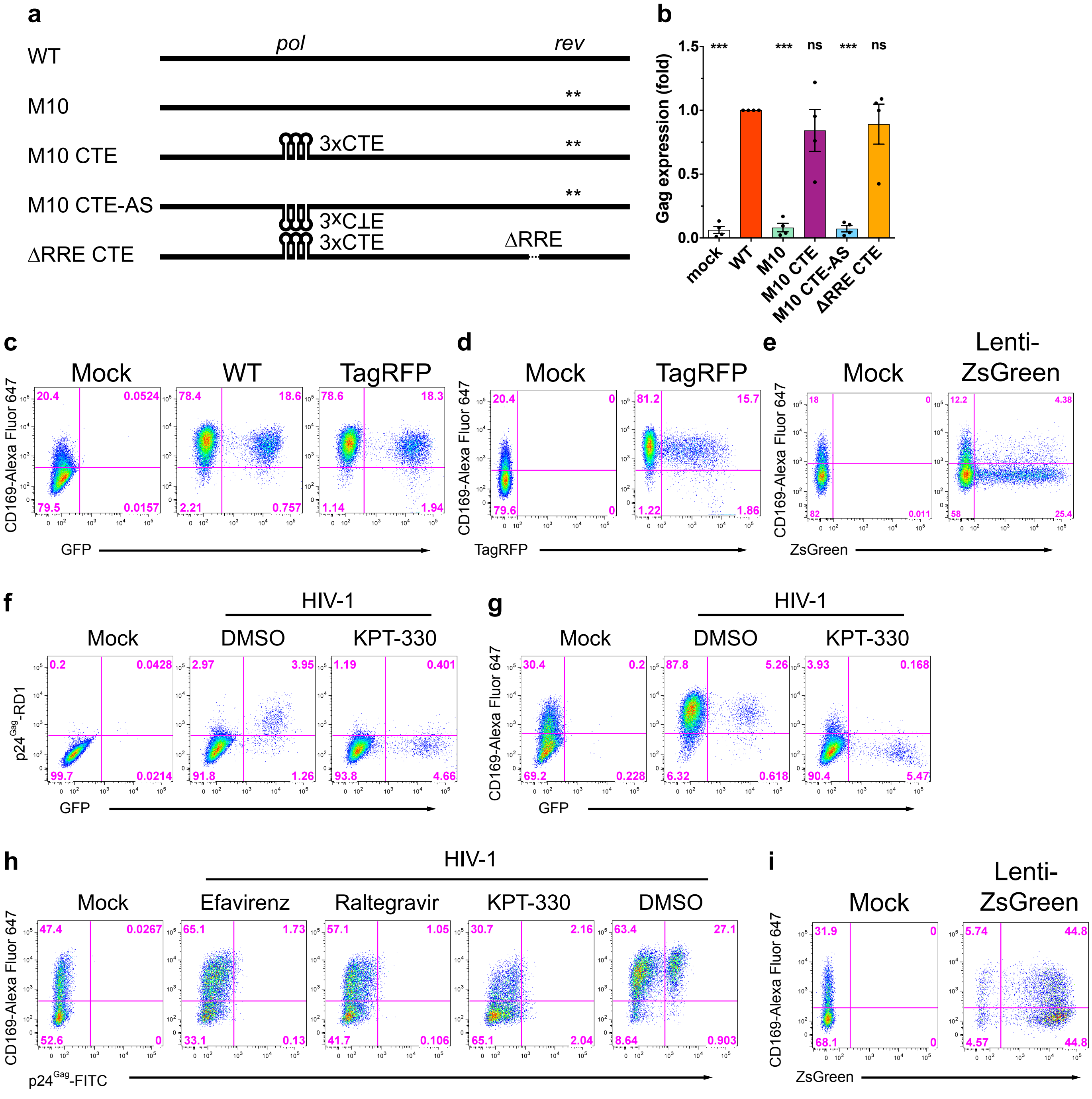

Supplementary Figure 3. Rev–CRM1 dependent HIV-1 icRNA export is important for sensing.
a, Schematic demonstration of HIV-1 mutants. *: position of mutation, CTE: constitutive transport element, RRE: Rev responsive element. **b**, Gag expression in infected MDMs with WT and Rev mutants (**Fig. 3a**) was quantitated and normalized to WT infection. Two-tailed p values were calculated using one-way ANOVA followed by the Dunnett's post-test to WT. ***: $p < 0.001$, ns: not significant. **c**, **d**, Representative flow cytometry profiles of MDMs transduced with the TagRFP mutant showing CD169 expression and GFP (in place of *nef*) (**c**) or TagRFP expression (in place of MA) (**d**) (6 dpi). **e**, Representative flow cytometry profiles of MDMs transduced with a lentiviral vector expressing ZsGreen driven by the human EF-1α promoter (6 dpi). **f**, Representative flow cytometry profiles of MDMs infected with HIV-1 in the absence (DMSO) or presence of CRM1 inhibitor KPT-330. KPT-330 inhibited $p24^{Gag}$ expression from unspliced RNA but did not inhibit GFP expression (in place of *nef*) from multiply spliced RNA (6 dpi). **g**, Representative flow cytometry profiles of MDMs infected with HIV-1 in the absence (DMSO) or presence of CRM1 inhibitor KPT-330 showing CD169 expression and viral infection (GFP) (6 dpi). **h**, Representative flow cytometry profiles of MDMs infected with HIV-1 in the absence (DMSO) or presence of CRM1 inhibitor KPT-330 showing CD169 expression and viral infection (GFP) (6 dpi). **i**, Representative flow cytometry profiles of MDDCs infected with HIV-1 in the absence (DMSO) or presence of HIV-1 inhibitors (EFZ: efavirenz, Ral: raltegravir) or CRM1 inhibitor KPT-330. MDMs were stained for CD169 and intracelluar $p24^{Gag}$ on day 3 post infection. **j**, Representative flow cytometry profiles of MDDCs infected with a lentiviral vector expressing ZsGreen and stained for CD169 on day 3 post infection.

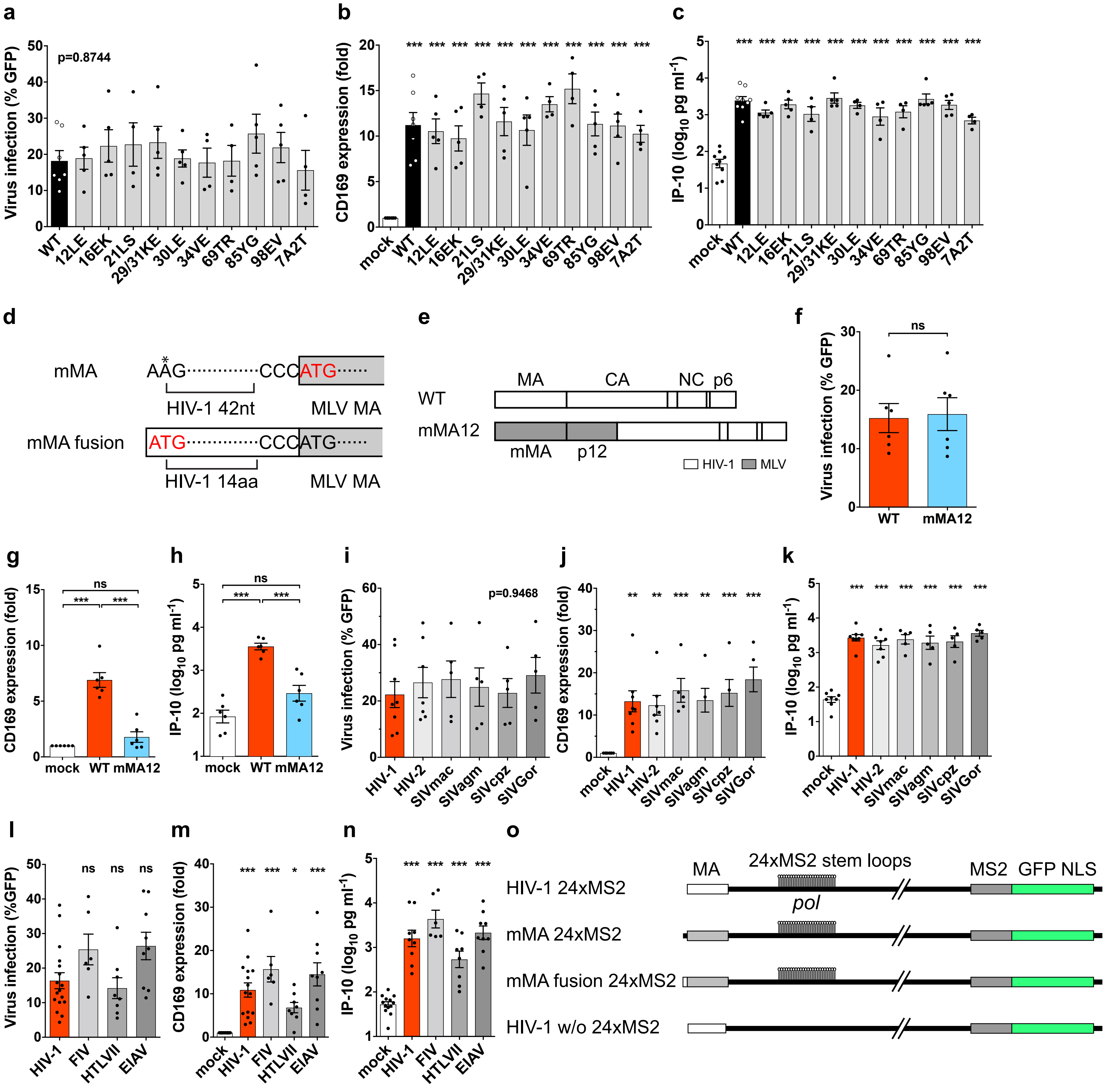

Supplementary Figure 4. Membrane targeting of viral icRNA is required for MDM activation.
a-c, Virus infection (GFP) (**a**), CD169 expression normalized to mock (**b**), and IP-10 production (**c**) in MDMs transduced with WT or indicated MA mutants lacking various MA functions (**Supplementary Table 1**) (6 dpi). **d**, Schematic demonstration of HIV-1 mutants encoding MLV MA. Both mutants contain HIV-1 MA sequences (42 nt) prior to the start codon for MLV MA. However, one (mMA) does not lead to expression of any of HIV-1 MA amino acids (aa) due to inactivation of the HIV-1 MA start codon (*), while the other (mMA-fusion) results in translation of first 14 aa of HIV-1 MA in frame with the MLV MA protein. **e**, Schematic demonstration of MLV/HIV-1 chimera, mMA12. **f-h**, Viral gene expression (GFP) (**f**), normalized CD169 expression (**g**), and IP-10 production (**h**) in MDMs infected (at MOI of 2) with WT or mMA12 (6 dpi). **i-k**, Viral gene expression (GFP) (**i**), CD169 expression normalized to mock (**j**), and IP-10 production (**k**) in MDMs transduced with WT or indicated chimeric viruses encoding MA from various primate lentiviruses (6 dpi). **l-n**, Viral gene expression (GFP) (**l**), CD169 expression normalized to mock (**m**), and IP-10 production (**n**) in MDMs transduced with WT or indicated chimeric viruses encoding MA from various lentiviruses and retroviruses (6 dpi). **o**, Schematic demonstration of HIV-1 mutants containing 24xMS2 stem loops in the *pol* orf and MS2-GFP-NLS fusion protein in place of the *nef* orf. The data shown are the means ± SEM and each symbol represents data obtained from cells derived from an independent donor. Two-tailed p values were calculated using one-way ANOVA followed by the Tukey-Kramer post-test (**g**, **h**) or the Dunnett's post-test (**b**, **c**, **j**, **k**, **l**, **m**, **n**), or a paired t-test (**f**), *: $p < 0.05$, **: $p < 0.01$, ***: $p < 0.001$, ns: not significant.

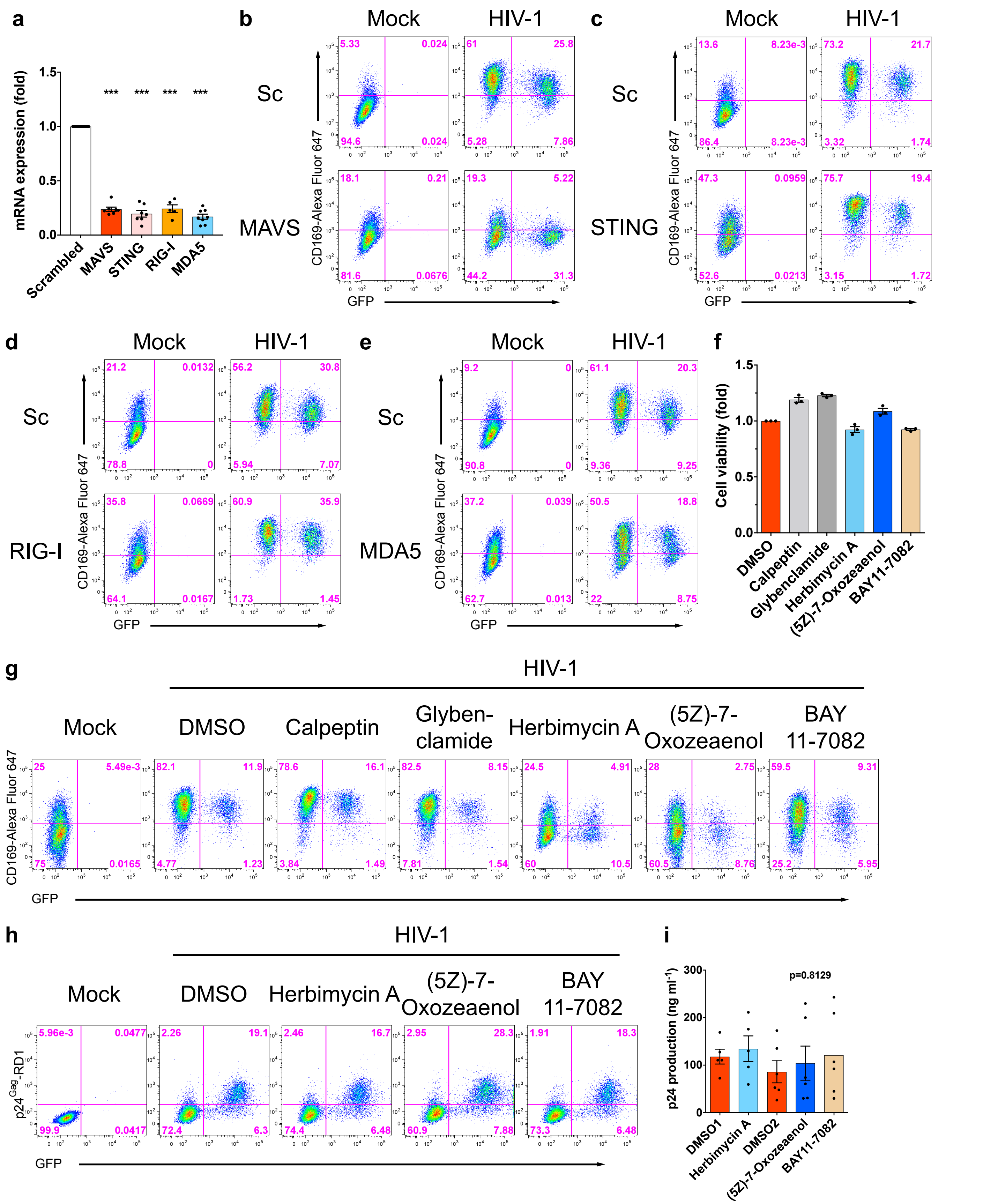

Supplementary Figure 5. MDM immune activation is initiated via a MAVS dependent pathway.
a, MDMs were transduced with lentiviral vectors expressing shRNA against indicated genes and mRNA levels of the target genes were quantitated with qRT-PCR. Each mRNA level was normalized to that of GAPDH and then normalized to that of scrambled-shRNA-transduced MDMs. **b, c**, Representative flow cytometry profiles of CD169 expression in MDMs transduced with either scrambled shRNA (Sc), shRNA against MAVS (**b**) or STING (**c**) and infected with LaiΔenvGFP/G (6 dpi). **d, e**, Representative CD169 expression profiles of MDMs transduced with scrambled shRNA (Sc), shRNA against RIG-I (**d**) or MDA5 (**e**) and infected with LaiΔenvGFP/G (6 dpi). **f**, MDMs were cultured for 6 days in the presence of the indicated inhibitors and cell viability was measured by MTT assay. The values were normalized to that of untreated (DMSO). **g**, Representative CD169 expression profiles of MDMs infected with HIV-1 in the presence of indicated inhibitors for 6 days. **h**, Representative flow cytometry profiles of MDMs infected with HIV-1 (GFP in place of *nef*) in the presence or absence (DMSO) of drugs showing intracellular $p24^{Gag}$ (y-axis) and GFP (x-axis) (6 dpi). **i**, MDMs were infected with HIV-1 and cultured with indicated drugs. The $p24^{Gag}$ contents in the supernatants harvested 6 dpi was measured. DMSO1: 0.1% DMSO, DMSO2: 0.01% DMSO. The data shown are the means ± SEM and each symbol represents data obtained from cells derived from an independent donor. Two-tailed p values were calculated using one-way ANOVA followed by the Dunnett's post-test. ***: $p < 0.001$.

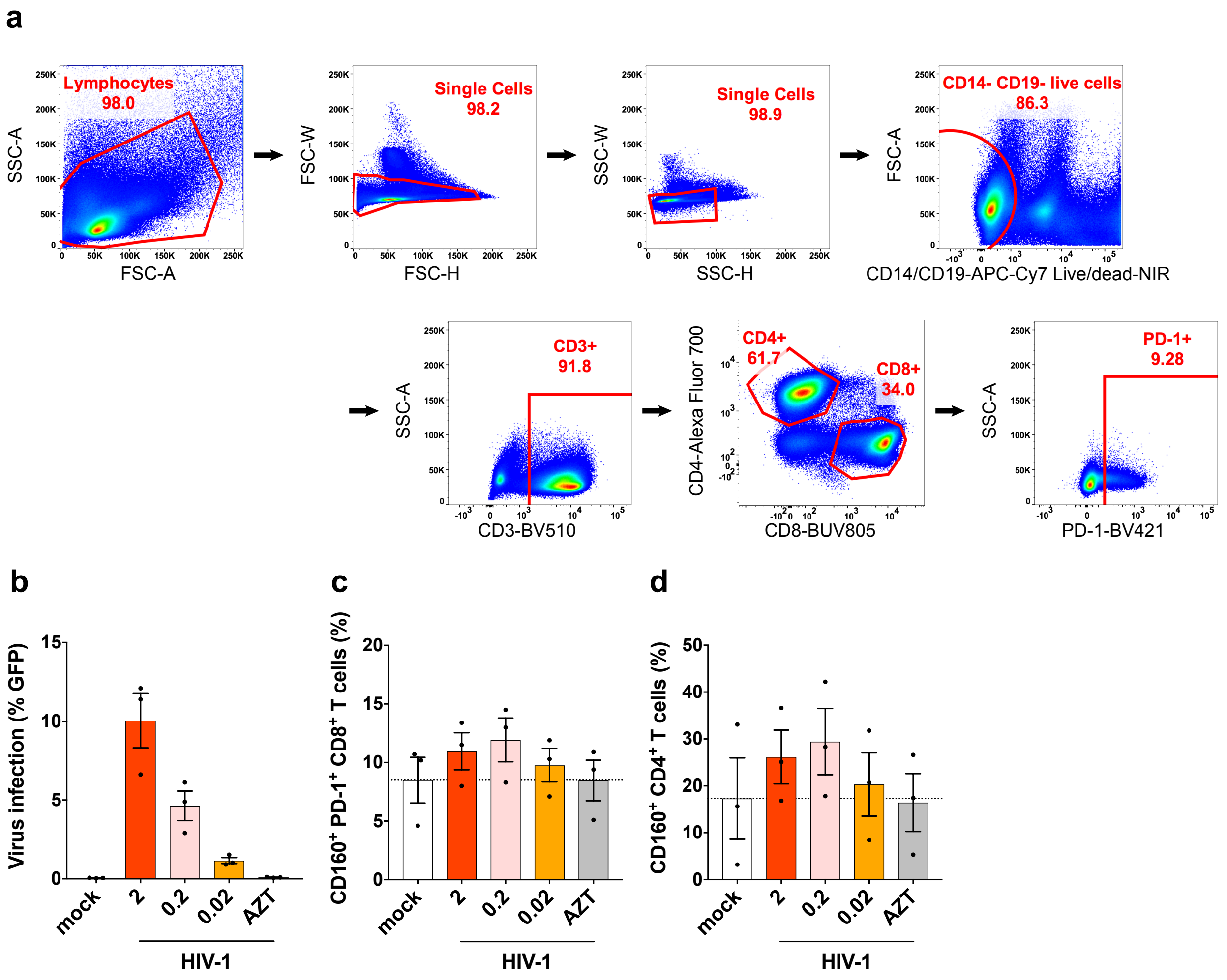

Supplementary Figure 6. HIV-1-infection-induced MDM activation results in expression of IRs on T cells and their functional impairment. **a**, Representative flow cytometry profiles to show the gating strategy for inhibitory receptor expression profiling and intracellular cytokine staining. **b**, MDMs were infected at various MOIs or in the presence of AZT, and GFP expression was measured with flow cytometry 6 days post infection. **c, d**, MDMs infected with HIV-1 at different MOIs were co-cultured with PBMCs, and the percentage of $CD160^{+}$ $PD\text{-}1^{+}$ $CD8^{+}$ T cells (**c**) and $CD160^{+}$ $CD4^{+}$ T cells (**d**) on day 5 post initiation of co-culture was quantitated by flow cytometry. The dotted lines indicate the background levels (mock).

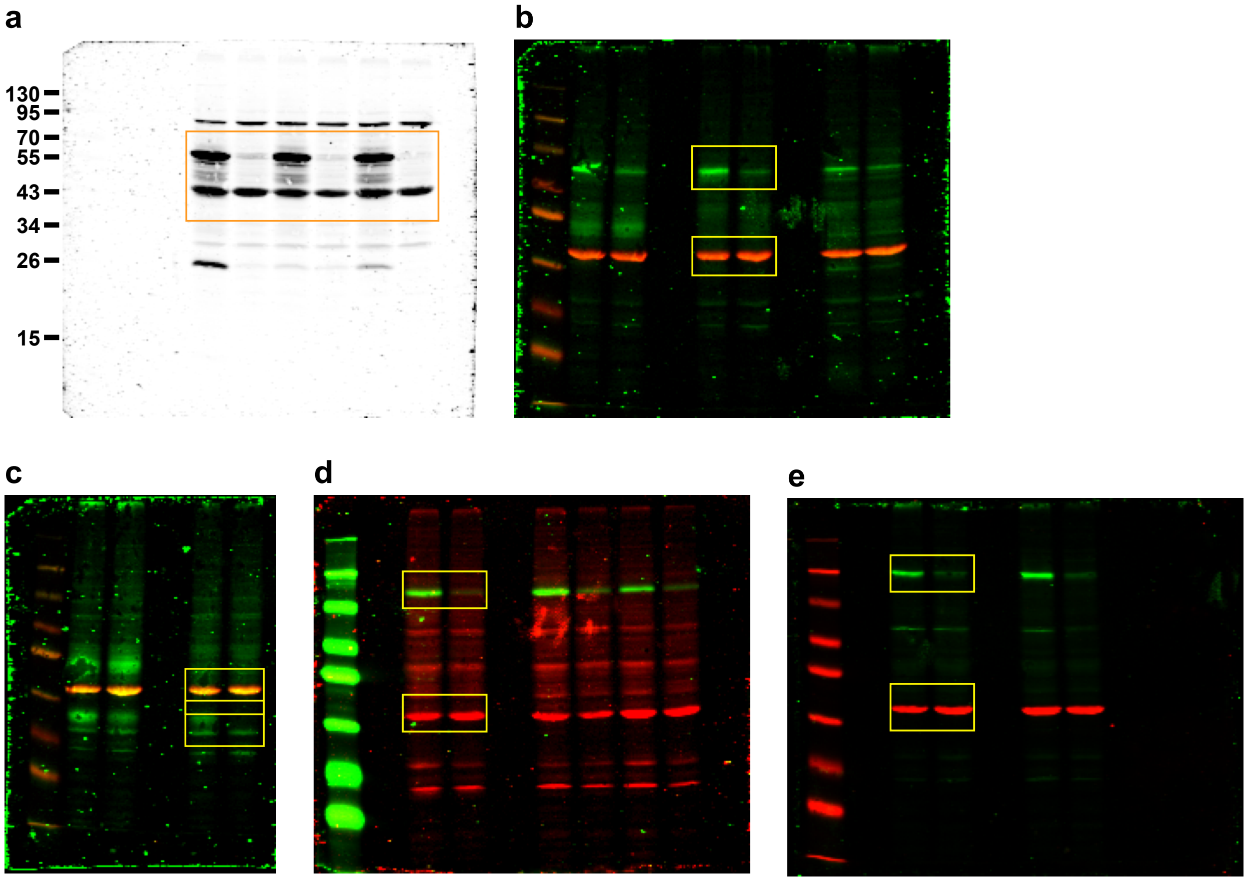

Supplementary Figure 7. Uncropped immunoblot images.
Uncropped immunoblot images for (**a**) Figure 3a, (**b**) Figure 5a, MAVS, (**c**) Figure 5a, STING, (**d**) Figure 5f, RIG-I and (**e**) Figure 5f, MDA5. Orange and yellow rectangles indicate where images were cropped.

Supplementary Table 1

Plasmid		Primer	Ref.
**M10**	F	GCCTCTTCAGCTACCACCGGATCTGAGACTTACTCTTGATTGTAAC	1
	R	GTTACAATCAAGAGTAAGTCTCAGATCCGGTGGTAGCTGAAGAGGC	
**CTE**	F	TTTTTGGATCCACTATAGGGCGAATTGAATTTAGCG	2
	R	GGATAACAAT TTCACACAGG AAACAGCTAT GAC	
**CTE AS**	F	TTTTTGCTAGCACTATAGGGCGAATTGAATTTAGCG	2
	R	TTTTTGGATCCCAGAATTAACCCTCACTAAAGGGAC	
**ΔMA**	F	TTTAAAGCTA GCCAGGTCAG CCAAAATTAC CCTATA	
	R	AAATTTGCTA GCTCTCGCAC CCATCTCTCT CCTT	
**ATG***	F	GCTAGAAGGAGAGAGAAGGGTGCGAGAGCGT	
	R	ACGCTCTCGCACCCTTCTCTCTCCTTCTAGC	
**HIV-1 + AatII**	F	AAAAAAGACGTCAGCCAAAATTACCCTATAGTGCAGAAC	
	R	CTGGGTTCGCATTTTGGACC	
**HIV-1-vec**	F	AGGCTAGAAGGAGAGCCATGGGTGCGAGAGCG	
	R	CGCTCTCGCACCCATGGCTCTCCTTCTAGCCT	
**HIV-1-vec-plus**	F	AGAGGAGCTC TCTCGACG	3
	R	AAAAAACCATGGGATCTAATTCTCCCCCGCTTAATAC	
**HIV-1 MA**	F	AAAAACCATGGGTGCGAGAGCGTC	
	R	AAAACCGGTCTTCCGACGTCGTAATTTTGGCTGACCTGGC	
**HIV-2 MA**	F	AAAAACCATGGGCGCGAGAAACTC	
	R	AAAACCGGTCTTCCGACGTCGTAATTTCCTCCCTTCTCGC	
**MLV MA**	F	AAAAACCATGGGCCAGACTGTTACC	4
	R	AAAACCGGTCTTCCGACGTCATAAAGGGAGGATCGAGGCG	
**SIVmac MA**	F	AAAAACCATGGGCGTGAGAAACTC	
	R	AAAACCGGTCTTCCGACGTCGTAATTTCCTCCTCTGCC	
**SIVagm MA**	F	AAAAACCATGGGTGCGAGTAACTCAG	
	R	AAAACCGGTCTTCCGACGTCGTAATTTTGTGATCCACCAC	
**SIVcpz MA**	F	AAAAACCATGGGTGCGAGAGC	
	R	AAACCGGTCTTCCGACGTCGTATAATCTACTTCCGCTAGG	
**SIVgor MA**	F	AAAAACCATGGGTGCGAGAGC	
	R	AAACCGGTCTTCCGACGTCATAGTTCTGACTTGTTTCAGG	
**FIV MA**	F	AAAAACCATGGGGAATGGACAGGG	
	R	AAAAAAGACGTCATATGCCTGTGGAGGGCCTTC	
**HTLVII MA**	F	AAAAACCATGGGACAAATCCACGGG	5
	R	AAAAAAGACGTCGAAGCATTGCGTGGTGG	
**EIAV MA**	F	AAAAACCATGGGAGACCCTTTGACATG	6
	R	AAAAAAGACGTCATATTCTTCAGAGGGCTCAGACTG	
**TagRFP**	F	AAAAACCATGGTGTCTAAGGGCGAAGAGC	
	R	AAAAAAGACGTCTCAATTAAGTTTGTGCCCCAGTTTGC	
**12LE**	F	GTATTAAGCGGGGGAGAAGAAGATCGATGGGAAAAAATTCG	7
	R	CGAATTTTTTCCCATCGATCTTCTTCTCCCCCGCTTAATAC	
**16EK**	F	GGGGAGAATTAGATCGATGGAAGAAAATTCGGTTAAGGCC	8
	R	GGCCTTAACCGAATTTTCTTCCATCGATCTAATTCTCCCC	
**21LS**	F	GATGGGAAAAAATTCGGTCAAGGCCAGGGGGAAAG	9
	R	CTTTCCCCCTGGCCTTGACCGAATTTTTTCCCATC	
**29/31KE**	F	GGGGGAAAGA AAAAATATGA ATTAGAACAT ATAGTATGGG C	10
	R	GCCCATACTA TATGTTCTAA TTCATATTTT TTCTTTCCCC C	
**30LE**	F	GGCCAGGGGGAAAGAAAAAATATAAAGAAAAACATATAGTATGGGC	7
	R	GCCCATACTATATGTTTTTCTTTATATTTTTTCTTTCCCCCTGGCC	
**34VE**	F	AAATTAAAACATATAGAATGGGCAAGCAGGGAGCTAGAAC	11
	R	GTTCTAGCTCCCTGCTTGCCCATTCTATATGTTTTAATTT	
**69TR**	F	CTACAACCATCCCTTCAGAGAGGATCAGAAGAACTTAG	12
	R	CTAAGTTCTTCTGATCCTCTCTGAAGGGATGGTTGTAG	
**85YG**	F	ATACAGTAGCAACCCTCGGTTGTGTGCATCAAAGGATAG	13
	R	CTATCCTTTGATGCACACAACCGAGGGTTGCTACTGTAT	
**98EV**	F	GATAGAGATAAAAGACACCAAGGTTGCTTTAGACAAGATAGAGG	12
	R	CCTCTATCTTGTCTAAAGCAACCTTGGTGTCTTTTATCTCTATC	
**7A2T**	F	GTTAGCGCCAGGGGGAGCGGCAGCATATACATTAACACATATAGTATGGGCAAGCAGGGAGC	14
	R	CCGCTCCCCCTGGCGCTAACGCAATTGCTTCCCATGCATCTAATTCTCCCCCGCTTAATACTGACG	
**MS2-GFP 5'**	F	TTTTTCCATGGCTTCTAACTTTACTCAGTTCGTTC	15
	R	CTCACCATGGTGGCGACCGGTGGGTCCGCGTAGATGCCG	
**MS2-GFP 3'**	F	TTCCCACCGGTCGCCACC	15
	R	TTTTTCTCGAGTTATACCTTTCTCTTCTTTTTTGGCTTG	

Supplementary Table 2

Gene	Sequence	Ref.
**MAVS**	ATGTGGATGTTGTAGAGATTC	Sigma (TRCN0000236031)
**STING**	GCCCGGATTCGAACTTACAAT	Sigma (TRCN0000163296)
**RIG-I**	CCAGAATTATCCCAACCGATA	Sigma (TRCN0000153712)
**MDA5**	CCAACAAAGAAGCAGTGTATA	Sigma (TRCN0000050849)

Supplementary Table 3

Gene	Forward	Reverse
**HIV-1 RNA**	TGTGTGCCCGTCTGTTGTGT	CTCTCCTTCTAGCCTCCGCT
**GAPDH**	CAAGATCATCAGCAATGCCT	AGGGATGATGTTCTGGAGAG
**IFNβ**	ATTCTAACTGCAACCTTTCG	GTTGTAGCTCATGGAAAGAG
**CD169**	GGCTGTTACGATGGTTTATGATG	AATCAAAGGCATCATTTTAGGGATA
**IP-10**	AAAGCAGTTAGCAAGGAAAG	TCATTGGTCACCTTTTAGTG
**ISG15**	TCCTGGTGAGGAATAACAAGGG	GTCAGCCAGAACAGGTCGTC
**ISG54**	GGTCTCTTCAGCATTTATTGGTG	TGCCGTAGGCTGCTCTCCA
**ISG56**	TAGCCAACATGTCCTCACAGAC	TCTTCTACCACTGGTTTCATGC
**Viperin**	TGGGTGCTTACACCTGCTG	GAAGTGATAGTTGACGCTGGTT
**Mx1**	GTTTCCGAAGTGGACATCGCA	CTGCACAGGTTGTTCTCAGC
**MAVS**	GTACCCGAGTCTCGTTTC	GCAGAATCTCTACAACATCC
**STING**	ACTGTGGGGTGCCTGATAAC	TGGCAAACAAAGTCTGCAAG
**RIG-I**	ATCCCAGTGTATGAACAGCAG	GCCTGTAACTCTATACCCATGTC
**MDA5**	GGCATGGAGAATAACTCATCAG	CTCTTCATCTGAATCACTTCCC

Supplementary References

1 Malim, M. H., Bohnlein, S., Hauber, J. & Cullen, B. R. Functional dissection of the HIV-1 Rev trans-activator--derivation of a trans-dominant repressor of Rev function. *Cell* **58**, 205-214 (1989).

2 Wodrich, H., Schambach, A. & Krausslich, H. G. Multiple copies of the Mason-Pfizer monkey virus constitutive RNA transport element lead to enhanced HIV-1 Gag expression in a context-dependent manner. *Nucleic acids research* **28**, 901-910 (2000).

3 Reed, M. *et al.* Chimeric human immunodeficiency virus type 1 containing murine leukemia virus matrix assembles in murine cells. *Journal of virology* **76**, 436-443 (2002).

4 Yamashita, M. & Emerman, M. Capsid is a dominant determinant of retrovirus infectivity in nondividing cells. *Journal of virology* **78**, 5670-5678, doi:10.1128/JVI.78.11.5670-5678.2004 (2004).

5 Shimotohno, K. *et al.* Complete nucleotide sequence of an infectious clone of human T-cell leukemia virus type II: an open reading frame for the protease gene. *Proceedings of the National Academy of Sciences of the United States of America* **82**, 3101-3105 (1985).

6 Patel, M., Giddings, A. M., Sechelski, J. & Olsen, J. C. High efficiency gene transfer to airways of mice using influenza hemagglutinin pseudotyped lentiviral vectors. *The journal of gene medicine* **15**, 51-62, doi:10.1002/jgm.2695 (2013).

7 Freed, E. O. & Martin, M. A. Virion incorporation of envelope glycoproteins with long but not short cytoplasmic tails is blocked by specific, single amino acid substitutions in the human immunodeficiency virus type 1 matrix. *Journal of virology* **69**, 1984-1989 (1995).

8 Joshi, A., Ablan, S. D., Soheilian, F., Nagashima, K. & Freed, E. O. Evidence that productive human immunodeficiency virus type 1 assembly can occur in an intracellular compartment. *Journal of virology* **83**, 5375-5387, doi:10.1128/JVI.00109-09 (2009).

9 Sherer, N. M., Swanson, C. M., Papaioannou, S. & Malim, M. H. Matrix mediates the functional link between human immunodeficiency virus type 1 RNA nuclear export elements and the assembly competency of Gag in murine cells. *Journal of virology* **83**, 8525-8535, doi:10.1128/JVI.00699-09 (2009).

10 Ono, A., Orenstein, J. M. & Freed, E. O. Role of the Gag matrix domain in targeting human immunodeficiency virus type 1 assembly. *Journal of virology* **74**, 2855-2866 (2000).

11 Freed, E. O. & Martin, M. A. Domains of the human immunodeficiency virus type 1 matrix and gp41 cytoplasmic tail required for envelope incorporation into virions. *Journal of virology* **70**, 341-351 (1996).

12 Tedbury, P. R., Ablan, S. D. & Freed, E. O. Global rescue of defects in HIV-1 envelope glycoprotein incorporation: implications for matrix structure. *PLoS pathogens* **9**, e1003739, doi:10.1371/journal.ppat.1003739 (2013).

13 Freed, E. O., Orenstein, J. M., Buckler-White, A. J. & Martin, M. A. Single amino acid changes in the human immunodeficiency virus type 1 matrix protein block virus particle production. *Journal of virology* **68**, 5311-5320 (1994).

14 Chukkapalli, V., Oh, S. J. & Ono, A. Opposing mechanisms involving RNA and lipids regulate HIV-1 Gag membrane binding through the highly basic region of the matrix domain. *Proceedings of the National Academy of Sciences of the United States of America* **107**, 1600-1605, doi:10.1073/pnas.0908661107 (2010).

15 Fusco, D. *et al.* Single mRNA molecules demonstrate probabilistic movement in living mammalian cells. *Current biology : CB* **13**, 161-167 (2003).
